# MS Annika 2.0 Identifies Cross-Linked Peptides in MS2–MS3-Based Workflows at High Sensitivity and Specificity

**DOI:** 10.1021/acs.jproteome.3c00325

**Published:** 2023-08-11

**Authors:** Micha J. Birklbauer, Manuel Matzinger, Fränze Müller, Karl Mechtler, Viktoria Dorfer

**Affiliations:** † Bioinformatics Research Group, University of Applied Sciences Upper Austria, Softwarepark 11, 4232 Hagenberg, Austria; ‡ Research Institute of Molecular Pathology (IMP), Vienna BioCenter (VBC), Campus-Vienna-Biocenter 1, 1030 Vienna, Austria; § Institute of Molecular Biotechnology (IMBA), Austrian Academy of Sciences, Vienna BioCenter (VBC), Dr. Bohr-Gasse 3, 1030 Vienna, Austria; ∥ Gregor Mendel Institute (GMI), Austrian Academy of Sciences, Vienna BioCenter (VBC), Dr. Bohr-Gasse 3, 1030 Vienna, Austria

**Keywords:** cross-linking, bioinformatics, search engine, MS3, XL-MS, protein-protein interaction, PPI

## Abstract

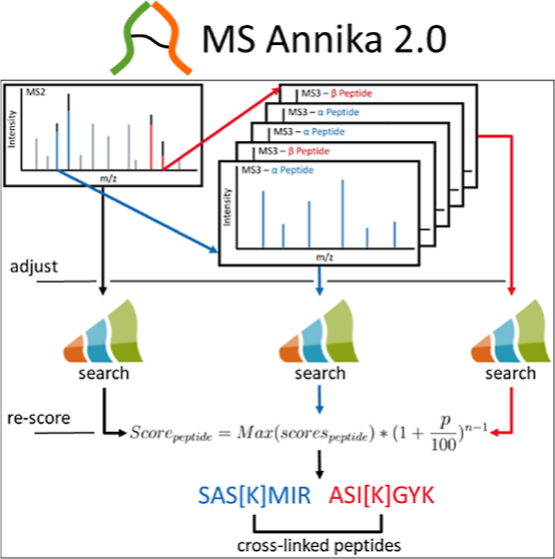

Cross-linking mass spectrometry has become a powerful tool for the identification of protein–protein interactions and for gaining insight into the structures of proteins. We previously published MS Annika, a cross-linking search engine which can accurately identify cross-linked peptides in MS2 spectra from a variety of different MS-cleavable cross-linkers. In this publication, we present MS Annika 2.0, an updated version implementing a new search algorithm that, in addition to MS2 level, only supports the processing of data from MS2–MS3-based approaches for the identification of peptides from MS3 spectra, and introduces a novel scoring function for peptides identified across multiple MS stages. Detected cross-links are validated by estimating the false discovery rate (FDR) using a target-decoy approach. We evaluated the MS3-search-capabilities of MS Annika 2.0 on five different datasets covering a variety of experimental approaches and compared it to XlinkX and MaXLinker, two other cross-linking search engines. We show that MS Annika detects up to 4 times more true unique cross-links while simultaneously yielding less false positive hits and therefore a more accurate FDR estimation than the other two search engines. All mass spectrometry proteomics data along with result files have been deposited to the ProteomeXchange consortium via the PRIDE partner repository with the dataset identifier PXD041955.

## Introduction

Cross-linking mass spectrometry (XLMS) has become a prominent tool in structural, molecular, and systems biology.^1^ XLMS is able to detect amino acid residues that are in close spatial proximity and therefore facilitating the structural analysis of proteins or protein complexes^2,3^ and capturing protein–protein interactions, potentially uncovering whole interactomes on a system-wide level.^4,5^ Various in-depth reviews that already exist not only highlight successful applications but also the limitations of XLMS.^6−10^ In a typical cross-linking experiment, the sample is incubated with a reagent, the cross-linker, that forms covalent bonds between reactive, surface-exposed amino acid side chains, which are within the cross-linker’s distance threshold. The sample is subsequently enzymatically digested (usually with trypsin) and optionally enriched for cross-linked peptides. Analysis is then carried out by liquid chromatography tandem mass spectrometry (LC–MS/MS) and cross-linked peptides and binding sites are identified from the resulting spectra by a cross-linking search engine.^11^

XLMS started out with studies on smaller proteins and protein complexes, using cross-linking reagents such as disuccinimidyl suberate or bis-(sulfosuccinimidly)suberate, that are still in use today.^12^ These reagents belong to the class of so-called non-cleavable cross-linkers since they do not break during fragmentation. Identification of peptides cross-linked with a non-cleavable cross-linker is complicated by the fact that only the complete mass of the cross-linked entity is known rather than the individual masses of the two cross-linked peptides.^13^ As a result, all possible pairs of peptides need to be considered during the search, quadratically increasing the search space which is often referred to as the *n*-squared problem.^14^ Estimation of the false discovery rate (FDR) is also more complex because the peptide identifications are interdependent on each other (a more detailed overview of FDR estimation in XLMS is given by Fischer and co-workers^15^). Especially the increase in search space and therefore runtime makes proteome-wide XLMS studies with non-cleavable cross-linkers challenging. These limitations led to the development of a new class of cross-linking reagents denoted as (MS-)cleavable cross-linkers, such as disuccinimidyl dibutyric urea (DSBU)^16^ and dissuccinimidyl sulfoxide (DSSO).^17^

In comparison to non-cleavable cross-linkers, cleavable cross-linkers incorporate an off-center labile moiety which can be cleaved during the fragmentation in the mass spectrometer, preferably at a lower collision energy before peptide backbone cleavage.^13,18^ Because cleavable cross-linkers break at an off-center position, they generate characteristic fragmentation patterns called doublets in the MS2 scan that allow the mass calculation of the two individual cross-linked peptides. As the name suggests, these doublets consist of two peaks that exhibit a cross-linker-specific mass difference (as shown in Figure S2). The two cross-linked peptides are often denoted as alpha and beta peptides, respectively; however, there is no clear consensus within the cross-linking community as to which of the two peptides should be the alpha and which the beta. Initial work by Schilling and co-workers^19^ proposes naming the longer peptide alpha and the shorter beta. For MS Annika, we have adopted the naming convention suggested by Kao and co-workers in the publication of DSSO^17^ that is widely used for cleavable cross-linkers, where the alpha peptide is the one associated with the doublet of lower *m*/*z*. Another advantage of cleavable cross-linkers is that the two cross-linked peptides can be selected for a third-stage tandem MS (MS3) scan, ideally acquiring an MS3 spectrum for each of the doublet peaks which contain the cross-linked peptides in linear form.

Even though the MS2 scan contains fragment ions of both cross-linked peptides, theoretically sufficient for identification of both peptides, usually an additional MS2 scan with a complimentary fragmentation method or doublet MS3 scans are required to obtain a satisfying sequence coverage for unambiguous cross-link identification.^20^ Alternatively, more recent studies have shown that stepped higher-energy C-trap dissociation (HCD)-based fragmentation methods are also performing well for cross-link identification.^20,21^ Using MS2–MS3 acquisition generally yields more cross-link identifications than traditional (non-stepped HCD) MS2- and MS2–MS2-based workflows while also providing higher sequence coverage^20^ and more accurate results for quantification than stepped HCD-based workflows.^22^ The advancements in acquisition methods and incorporation of ion-mobility^23,24^ or FAIMS filtering^25^ have further improved cross-link identification rates, and XLMS using cleavable cross-linkers is nowadays being used for experiments up to system wide level.^26^

The increasing number of cross-linking reagents, different acquisition methods, and various experimental setups led to the implementation of over 20 different software tools (usually denoted as cross-linking search engines) from different research groups specializing in the identification and validation of cross-linked peptides.^27−34^ It should also be noted here that most traditional database search engines for the identification of linear peptides can be used to detect cross-linked peptides specifically in MS3 scans, in that regard Protein Prospector^35^ has been successfully applied, as shown in recent publications.^36,37^ However, combining the identified peptides to cross-links via their precursor MS2 spectra and subsequent validation is a substantial part of cross-link identification that still needs to be done manually or by another software afterward. Cross-linking search engines typically incorporate all of these steps with very little need for manual work.

To our knowledge, only two cross-linking search engines support data from MS2–MS3 acquisition, namely, MaXLinker^29^ and XlinkX.^34^ However, both tools have significant drawbacks: MaXLinker requires multiple manual pre-processing steps that complicate data analysis while also often identifying substantially less cross-links than XlinkX. On the other hand, XlinkX usually identifies a high number of unique cross-links, but it suffers from poor FDR estimation, often highly underestimating the actual FDR and therefore identifying a lot of false positive cross-links.^13^ This highlights the need for a cross-linking search engine that is capable of reliably identifying cross-linked peptides from MS3 spectra while providing accurate FDR estimates.

In this work, we present MS Annika 2.0, a new version of our cross-linking search engine that we previously published.^38^ The updated MS Annika 2.0 now supports data from any MS2–MS3-based cross-linking workflow and introduces a new algorithm for identifying cross-linked peptides from MS3 spectra that reliably detects high numbers of cross-links while providing accurate FDR estimates. MS Annika 2.0 is available free of charge for Thermo Scientific Proteome Discoverer versions 2.5, 3.0, and 3.1 at https://ms.imp.ac.at/index.php?action=ms-annika.

## Methods

**Figure 1:**
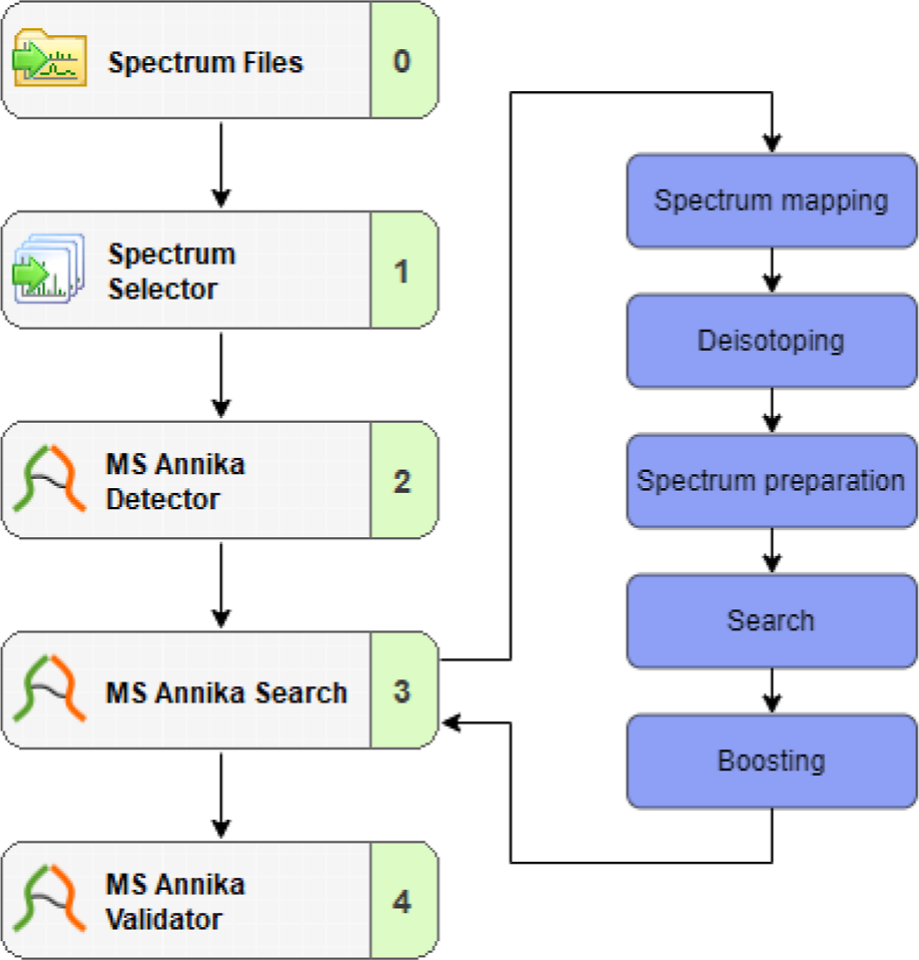
Exemplary workflow of an MS2–MS3 search with MS Annika 2.0 in Proteome Discoverer (left) and the different stages of the MS2–MS3 search algorithm (right). MS3 spectra are mapped to their respective precursor MS2 spectra to detect the peptide’s cross-link modification → the cross-link doublet peaks are deisotoped for calculating the peptide’s monoisotopic mass → spectra are adjusted and then searched with MS Amanda^39^ to identify the cross-linked peptides → scores of hits of the same precursor that appear in more than one MSn scan are boosted to reflect the increased confidence.

Our newly developed search algorithm for MS2–MS3-based cross-linking workflows builds upon the characteristic fragmentation patterns created by MS-cleavable cross-linkers in MS2 spectra as well as the product MS3 spectra containing the cross-linked peptides in linear form. The detection of these characteristic fragmentation patterns in the MS2 spectra is facilitated by the MS Annika Detector node that we described in our previous MS Annika publication.^38^ In order to adapt MS Annika 2.0 for MS2–MS3-based workflows and identify cross-linked peptides also from MS3 spectra, every MS3 spectrum is first mapped to its corresponding precursor peak in the associated MS2 spectrum. This mainly serves two functions: (1) to verify that the MS3 spectrum indeed originates from a cross-linked peptide and (2) to determine the cross-linker modification attached to that peptide. Subsequently, deisotoping of the cross-link peaks in the MS2 spectrum is performed to calculate the monoisotopic mass of the cross-linked peptides. Then the MS2 and MS3 spectra are adjusted for identification by our in-house developed search engine, MS Amanda,^39^ that identifies both cross-linked peptides. If the same peptide is found in an MS2 spectrum and one or more of the associated product MS3 spectra, its confidence is boosted using a novel scoring function. All identified peptides are then grouped to cross-link spectrum matches (CSMs) and validated using a target-decoy approach to estimate the FDR. Similarly, the CSMs are grouped to cross-links and validated again using the same target-decoy strategy. A schematic workflow of the MS2–MS3 search in MS Annika 2.0 is depicted in Figure 1.

### MS3 Spectrum to MS2 Doublet Peak Mapping

The first step in our MS3 search algorithm is, as aforementioned, creating a mapping connecting MS2 doublet peaks to their corresponding MS3 product spectra. The MS Annika detector node searches for cross-linker-specific fragmentation patterns in the MS2 spectrum and annotates peaks that are likely to originate from cross-linked peptides as doublet peaks: either alpha or beta, describing if it belongs to the first or second peptide, and light or heavy depending on the attached cross-linker modification. MS Annika 2.0 is then searching for any product MS3 spectra of that MS2 scan within a 5 min retention time window after being recorded by looking for MS3 scans that share the same MS1 precursor mass as the MS2 scan. All eligible MS3 spectra are attempted to be matched to one of the doublet peaks (or peaks of the same isotopic envelope) using a given tolerance. If a match is found, key information about the peptide contained in the MS3 can be deducted: first, the accurate monoisotopic mass of the cross-linked peptide can be determined (if the isotopic distribution is present in the precursor MS2 spectrum). Second, the cross-linker modification is given by the doublet peak annotation as the peptide originating from the lighter peak carries the short arm of the cleavable cross-linker while the heavier peak carries the long arm. Third, by considering the distance between the two peaks in the doublet, we can detect if the cross-linker has undergone any additional modifications, which is important for an accurate calculation of the unmodified peptide’s mass (see also Figure S3). Furthermore, another advantage of this matching process is the verification that an MS3 spectrum indeed stems from a peak of a cross-linked peptide. Even though mass spectrometers are tuned to select one of the doublet peaks for subsequent fragmentation and recording of an MS3 scan, it has been shown that they sometimes fail to do so and analyze another close (in terms of *m*/*z*) but non-cross-linked peak instead.^21^ Therefore, rather than searching all MS3 spectra and potentially identifying a lot of false positives, we only search those whose precursor MS2 spectrum shows cross-link evidence and where the precursor peak in the MS2 spectrum is part of a cross-link doublet.

### Spectrum Preparation and Search

The second step in our MS3 search algorithm is preparing both MS2 and MS3 spectra for search with our in-house developed database search engine, MS Amanda,^39^ to identify the cross-linked peptides. Deisotoping of the doublet peaks in the MS2 spectra is performed to determine the monoisotopic masses of the peptides since the instrument does not always select the monoisotopic peak for the MS3 scan. Furthermore, for identification, the algorithm tries to infer the charge of the peptide from the isotopic distribution of the MS2 or MS3 scan, or if it cannot be inferred, all charge states up to a user-definable limit (by default 4) are considered. The precursor *m*/*z* of any candidate MS3 spectrum is then adjusted to that of the monoisotopic precursor peak minus the *m*/*z* of the cross-linker modification(s), and the precursor charge is set to the detected charge state. Moreover, MS Annika does not only consider the short and long fragments of the cross-linker as modifications but also any potential mass changes due to alternative cleavages or losses (see DSSO^17^/DSBSO^40^ and Figure S3). The precursor masses (mass of the peptide without the cross-linker) of the MS3 spectra are calculated as the following
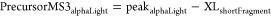
1
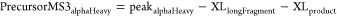
2
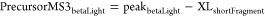
3
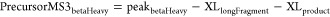
4where peak is the mass of the monoisotopic doublet peak in the associated MS2 precursor spectrum and XL_product_ is the mass of any potential change that the cross-linker fragment underwent.

Additionally, if only one of the peptides is captured in an MS3 scan, the MS2 scan is used for the identification of the other peptide, where the precursor mass of the MS2 is adjusted in the same manner. Optionally, this is also carried out if there are MS3 scans for both peptides. Moreover, the approach described in our previous publication^38^ is still utilized to identify cross-linked peptides from MS2 spectra; on the one hand, to gather more evidence for identifications and on the other hand, to cover all cases where none of the peptides are featured in one of the MS3 scans.

MS2 and MS3 spectra are then searched with MS Amanda^39^ to identify the cross-linked peptides, considering individual mass tolerances for the different spectra.

### Peptide Hit Pooling and Boosting

In MS2–MS3-based workflows, the same peptide can often be identified in the MS2 scan and in one or more of the corresponding product ion MS3 scans. If so, there is more evidence that the identified peptide is correct, which should be reflected in the peptide’s identification score. We therefore designed a new scoring function that boosts the peptide’s identification score based on its maximum MS Amanda score^39^ and the number of MSn scans in which it was identified. The scoring function is defined as the following
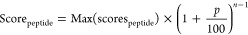
5where scores_peptide_ is a vector of all MS Amanda scores of the given peptide for an MS2 scan and its corresponding MS3 scans. Variable *n* is the number of unique corresponding MSn scans the peptide was identified in and *p* is the boost parameter that defines how much a hit is boosted.

For the choice of *p*, we tested several distinct values ranging from 0 (not boosting) to 100 (doubling the maximum score for every additional scan) on the dataset by Matzinger and co-workers^26^ that we describe later. By default, *p* is set to 20 which we validated to perform well across all tested datasets featuring various cross-linkers, acquisition methods and fragmentation methods.

### Validation

The two identified cross-linked peptides are then combined into one CSM that associates the two peptides with the MS2 spectrum that contains them (independently of whether the identifications are from the MS2 or MS3 scan). Since the same pair of peptides can usually be found in more than one spectrum, several CSMs of the same peptide pair are then grouped into one cross-link. Scoring and validation of CSMs and cross-links in the MS2–MS3 search works exactly the same as in the MS2 search: the score of the CSM is the smaller MS Amanda score of the two peptides and the score of the cross-link is the maximum score of all CSMs that form the cross-link. For FDR estimation, we employ a two-step validation approach, first on the CSM level and subsequently on the cross-link level, using a target-decoy strategy where decoys are generated by reversing the database. A more detailed description of our validation algorithm is given in our previous publication of the MS2 search of MS Annika.^38^

### Description of Datasets

For the evaluation of MS Annika 2.0, we used five datasets from different independent groups covering a range of cross-linkers, mass analyzers, acquisition methods and fragmentation methods, as well as various cross-linking workflow designs and use cases to get an accurate representation of real-world performance. A complete overview of used datasets is shown in Table 1.

**Table 1:** Datasets Used for Evaluating the Performance of the MS2–MS3 Search Algorithm of MS Annika 2.0

name	MS2/MS3 acquisition	MS3 mass analyzer	cross-linker	PRIDE ID
dataset A	CID/CID	ion trap/Orbitrap	DSSO/DSBSO	PXD041955
dataset B	CID/CID	Orbitrap	DSSO	PXD029252^26^
dataset C	CID(+EThcD)/CID	ion trap	DSSO	PXD014337^13^
dataset D	SCE/CID	ion trap	DSSO	PXD031114^22^
dataset E	CID/HCD	ion trap	DSSO	PXD026191^41^

For the first dataset—from hereon denoted as dataset A—we used a synthetic peptide library that we published previously^26^ and that was specifically designed for benchmarking cross-linking workflows and search engines. This peptide library features a total of 141 peptides from 38 different proteins of the *E. coli* ribosomal complex with a theoretical limit of 1018 cross-links that can be found. The peptides are split into groups of 6–10 peptides and cross-linked groupwise, allowing the calculation of the experimentally validated FDR after identification since it is known which peptides can be cross-linked. Specifically, any cross-links between peptides of different groups or non-synthesized peptides are false positives. For this publication, we re-analyzed the synthetic peptide library using four additional MS2–MS3-based cross-linking experiments. The library was cross-linked once with DSSO and once with DSBSO, and samples were analyzed on an Orbitrap Eclipse (Thermo Scientific) using collision-induced dissociation (CID) acquisition mode for MS2 and MS3 spectra. MS3 scans were recorded once using the Orbitrap and once using the ion trap, which allowed us to compare the performance of MS Annika 2.0 not only across different cross-linkers but also across different mass analyzers. All mass spectrometry proteomics data have been deposited to the ProteomeXchange consortium via the PRIDE partner repository^42^ with the dataset identifier PXD041955.

Additionally, we tested MS Annika 2.0 on the data that we published along with the synthetic peptide library,^26^ from hereon denoted as dataset B. Similar to dataset A, the peptide library was cross-linked with DSSO and analyzed on an Orbitrap Eclipse (Thermo Scientific) using CID acquisition for MS2 and MS3 scans. Both MS2 and MS3 spectra were recorded in the Orbitrap at a resolution of 30 and 15 K, respectively. The sample for this workflow was analyzed in triplicates—in contrast to dataset A which was not replicated. The dataset is available via the PRIDE public repository^42^ using the identifier PXD029252.

The third dataset that we used for benchmarking is the synthetic peptide library by Beveridge and co-workers,^13^ from hereon denoted as dataset C. Analogous to our synthetic peptide library of the ribosomal complex, their peptide library was also explicitly developed for evaluating cross-linking workflows and search engines, which allows us to calculate an experimentally validated FDR and assess how accurate a search engine’s FDR estimation is. The peptide library consists of 95 peptides from *S. pyogenes* Cas9 that were divided into 12 groups and cross-linked within their groups, yielding a theoretical limit of 426 cross-links that can be identified. Again, the premise is that any identified cross-link that is composed of two peptides of different groups or non-synthesized peptides is a false positive. Beveridge and co-workers applied their peptide library to two different MS2–MS3-based cross-linking workflows using DSSO with samples analyzed on an Orbitrap Fusion Lumos (Thermo Scientific). In the first workflow, MS2 and MS3 scans were acquired using CID fragmentation, while in the second workflow, an additional MS2 scan was recorded using electron-transfer/higher-energy collision dissociation (EThcD). In both workflows, MS3 scans were recorded in the ion trap. The dataset is available via the PRIDE public repository using the identifier PXD014337.

The fourth dataset was established by Ruwolt and co-workers^22^ for optimizing tandem mass tag-based quantification of protein–protein interactions in XLMS. We refer to this dataset from hereon as dataset D. As part of their optimization process, Ruwolt and co-workers compared different acquisition methods for cross-link identification, where one of the methods was an MS2–MS3-based workflow that we re-analyzed to benchmark our new MS3 search. In this workflow, they cross-linked HEK293T cell lysates with DSSO and prepared three strong cation exchange (SCX) fractions, which they analyzed using an Orbitrap Fusion Lumos with FAIMS Pro device (both Thermo Scientific) using stepped HCD for MS2 (Orbitrap) and CID for MS3 (ion trap) acquisition. Samples were analyzed in two technical replicates, and the data is available via the PRIDE public repository using the identifier PXD031114.

The fifth and final dataset—from hereon denoted as dataset E—that we used for testing MS Annika 2.0 is from a cross-linking experiment for the structural investigation of the non-structural protein 7 (NSP7) and non-structural protein 8 (NSP8) complex of SARS-CoV-2 by Courouble and co-workers.^41^ Specifically, we analyzed data from their NSP8 MS2–MS3 experiment, where they cross-linked NSP8 with DSSO and analyzed the sample on an Orbitrap Fusion Lumos (Thermo Scientific) using CID MS2 (Orbitrap) and HCD MS3 (ion trap) acquisition. The dataset consists of technical triplicates and can be retrieved via the PRIDE public repository using dataset identifier PXD026191.

### Data Acquisition

Except for dataset A, all data was retrieved via the PRIDE public repository^42^ using the respective identifiers. For dataset A, the samples were prepared as given in our publication.^26^ In order to acquire MS3 scans from the Orbitrap and the ion trap, the samples were analyzed on an Orbitrap Eclipse Tribrid mass spectrometer with a FAIMS pro interface (both Thermo Scientific). The Orbitrap Eclipse was operated in data-dependent mode using a full scan (*m*/*z* range 375–1500, a nominal resolution of 120 K, and an automatic gain control (AGC) target value of 4 × 10^5^). MS2 scans were acquired in the Orbitrap using CID fragmentation at a collision energy of 25, an isolation width of 1.2 *m*/*z*, a resolution of 30 K, and an AGC target value of 5 × 10^4^. Precursor ions selected for fragmentation (±10 ppm) were put on a dynamic exclusion list for 25 s. MS3 scans were triggered by the cross-linker-specific mass difference (DSSO: 31.9721 Da and DSBSO: 182.0071 Da) and either acquired in the Orbitrap or in the ion trap, depending on the experiment, using CID fragmentation at a collision energy of 35 and an isolation width of 2.0 *m*/*z*. For MS3 scans recorded in the Orbitrap, the resolution was set to 15 K, a maximum injection time of 22 ms, and an AGC target value of 2 × 10^4^. For MS3 scans recorded in the ion trap, the ion trap was operated in rapid mode with a maximum injection time of 150 ms and an AGC target value of 4 × 10^3^. The compensation voltage for FAIMS was set to −55 V. The MS3 acquisition was designed as described by Wheat and co-workers.^36^ All mass spectrometry data along with our result files have been deposited to the ProteomeXchange consortium via the PRIDE partner repository^42^ with the dataset identifier PXD041955.

### Data Processing and Evaluation

To assess the performance of our MS2–MS3 search algorithm, we compared MS Annika 2.0 to XlinkX^34^ and MaXLinker^29^ which are—to our knowledge—the only other two cross-linking search engines capable of MS2–MS3 searches. The metrics that we used to compare the different search engines were, on the one hand, the total number of unique cross-link identifications at an estimated 1% FDR and, on the other hand, how close the estimated FDR was to the experimentally validated FDR for datasets where this calculation was possible. We applied all search engines to the datasets mentioned previously.

We analyzed the datasets using MS Annika 2.0 (version 1.1.3, Proteome Discoverer version 2.5.0.400), XlinkX (version 2.5, Proteome Discoverer version 3.0.0.757), and MaXLinker (version 1.0.0). For searches with MS Annika 2.0 and XlinkX, RAW files were loaded into Proteome Discoverer, and MS Annika 2.0 and XlinkX were used as nodes within the software. We employed the workflow design proposed by XlinkX for both XlinkX and MS Annika 2.0. Mass spectra were first searched with a traditional database search engine such as Sequest HT (version 2.0.0.26, Proteome Discoverer version 3.0.0.757)^43^ for XlinkX workflows or MS Amanda (version 2.5.0.16129, engine version 2.0.0.16129, Proteome Discoverer version 2.5.0.400)^39^ for MS Annika 2.0 workflows for the purpose of identifying linear peptides. Subsequently, spectra with highly confident identifications of a linear peptide were filtered out and not considered for the cross-link search.

For searches with MaXLinker, we followed the steps given in their publication.^29^ Spectra were converted to MGF using the Spectrum Exporter node in Proteome Discoverer 2.5 (version 2.5.0.400) and to DTA format using the Spectrum Exporter node in Proteome Discoverer 2.1 (version 2.1.0.81). The Sequest HT searches necessary for MaXLinker for the identification of cross-linked peptides were carried out using Sequest HT (version 2.0.0.24) and validated using Percolator (version 3.05.0),^44^ both in Proteome Discoverer 2.5 (version 2.5.0.400).

Wherever possible, we used comparable settings across the different search engines for parameters which they all share, for example, precursor mass tolerance and fragment mass tolerance. We applied a precursor mass tolerance of 5–10 ppm, a fragment mass tolerance of 10–20 ppm for spectra recorded in the Orbitrap, and a 0.5 Da fragment mass tolerance for spectra recorded in the ion trap. Any search engine-specific parameters were set to the defaults recommended by the developers or to values used by the authors of the datasets if they were given. Moreover, for the data of the synthetic peptide libraries, we attempted to identify the parameters that yielded the maximum number of unique cross-link identifications at an experimentally validated FDR that is as close as possible to the estimated FDR. A complete overview of the applied search settings is given in the Supporting Information and shown in Table S1, the applied workflow design of the search is depicted in Figure S1.

For datasets A and B, we used the FASTA database provided in the respective PRIDE repository in combination with 116 sequences of common contaminants for search. For the datasets by Beveridge and co-workers (dataset C), we used the FASTA database given in the Supporting Information of their publication,^13^ which featured the sequence of *S. pyogenes* Cas9 and common contaminant proteins. Furthermore, for searching the datasets by Ruwolt and co-workers (dataset D), we used the FASTA database given in their PRIDE repository, including 5812 proteins from human UniProt/Swiss-Prot. We additionally added the proteome of *E. coli* (strain K12, proteome ID UP000000625, retrieved 03.10.2022) for better FDR control. In order to identify cross-links in the NSP8 datasets by Courouble and co-workers (dataset E), we used the reference sequence of NSP8 of SARS-CoV-2 from the National Center for Biotechnology Information (NCBI) with the identifier YP_009725303.1 (retrieved 12.10.2022).

Since MaXLinker only supports FASTA files in a specific format, all FASTA files used for analysis with MaXLinker were pre-processed with an in-house-developed Python script. To facilitate the comparison between the different tools, all cross-links identified by MaXLinker were manually grouped by sequence and cross-linker position to be consistent with the grouping that is applied by XlinkX and MS Annika 2.0. Finally, MaXLinker result files were converted to resemble the same structure as MS Annika 2.0 result files if needed to make downstream analysis easier as not all tools support MaXLinker files as input. We have published all the scripts that we developed as part of our analysis and which we imagine will increase the usability of MaXLinker for other researchers on GitHub via the repository https://github.com/hgb-bin-proteomics/MaXLinker_extensions.

Results of the synthetic peptide libraries (datasets A, B, and C) were post-processed using the tool IMP-X-FDR (version 1.1.0)^26^ that can calculate the experimentally validated FDR. The premise for FDR calculation is that peptides are cross-linked groupwise, and therefore any cross-link consisting of peptides that are not within the same group or cross-links involving non-synthesized peptides are considered false positives. The experimentally validated FDR is then calculated as the fraction of false positive cross-links divided by the number of all identified cross-links. Furthermore, for dataset D, we validated the identified cross-links, on top of the FDR estimation by the search engines, by using a strategy well established in traditional database search, which is usually referred to as entrapment search.^45^ Sequences of an organism not contained within the analyzed sample are added to the database for search in order to estimate the FDR among the target hits after target-decoy validation. Several publications have shown that this can also be applied to XLMS for validating cross-linking results.^5,46,47^ We used the proteome of *E. coli* for entrapment as there was no *E. coli* in the sample, consequently, any identification containing an *E. coli* peptide was considered a false positive hit. For validation, we therefore post-processed MS Annika 2.0, MaXLinker, and XlinkX results by filtering out the lowest scoring cross-link until less than 1% of identified cross-links contained an *E. coli* peptide.

### Visualization

We have developed several new exporters to visualize MS Annika 2.0 results with tools commonly used in XLMS like xiNET,^48^ xiVIEW,^49^ and PyMOL (using the plugin PyXlinkViewer^50^). The exporters are written in Python and can either be used from within Proteome Discoverer or as standalone scripts. PDB files are read using biopandas^51^ and in order to map cross-links to protein structures, the sequence alignment methods from biopython^52^ are used. All exporters are open-source and freely available on GitHub via the repository https://github.com/hgb-bin-proteomics/MSAnnika_exporters.

Visualizations of the cross-linked NSP7-NSP8 complex that are shown in Figure S13 are created in ChimeraX (version 1.4)^53^ using the plugin XMAS (version 1.1.2).^54^

## Results

We herein present the results achieved with our new MS2–MS3 search approach on the previously mentioned datasets. We show that MS Annika 2.0 yields high numbers of unique identifications and accurate FDR estimates in benchmark experiments with synthetic peptide libraries as well as in real-world cross-linking experiments.

### MS Annika 2.0 Provides High Numbers of True Cross-link Identifications and Realistic FDR Estimates for Ion Trap and Orbitrap MS3 Data

**Figure 2:**
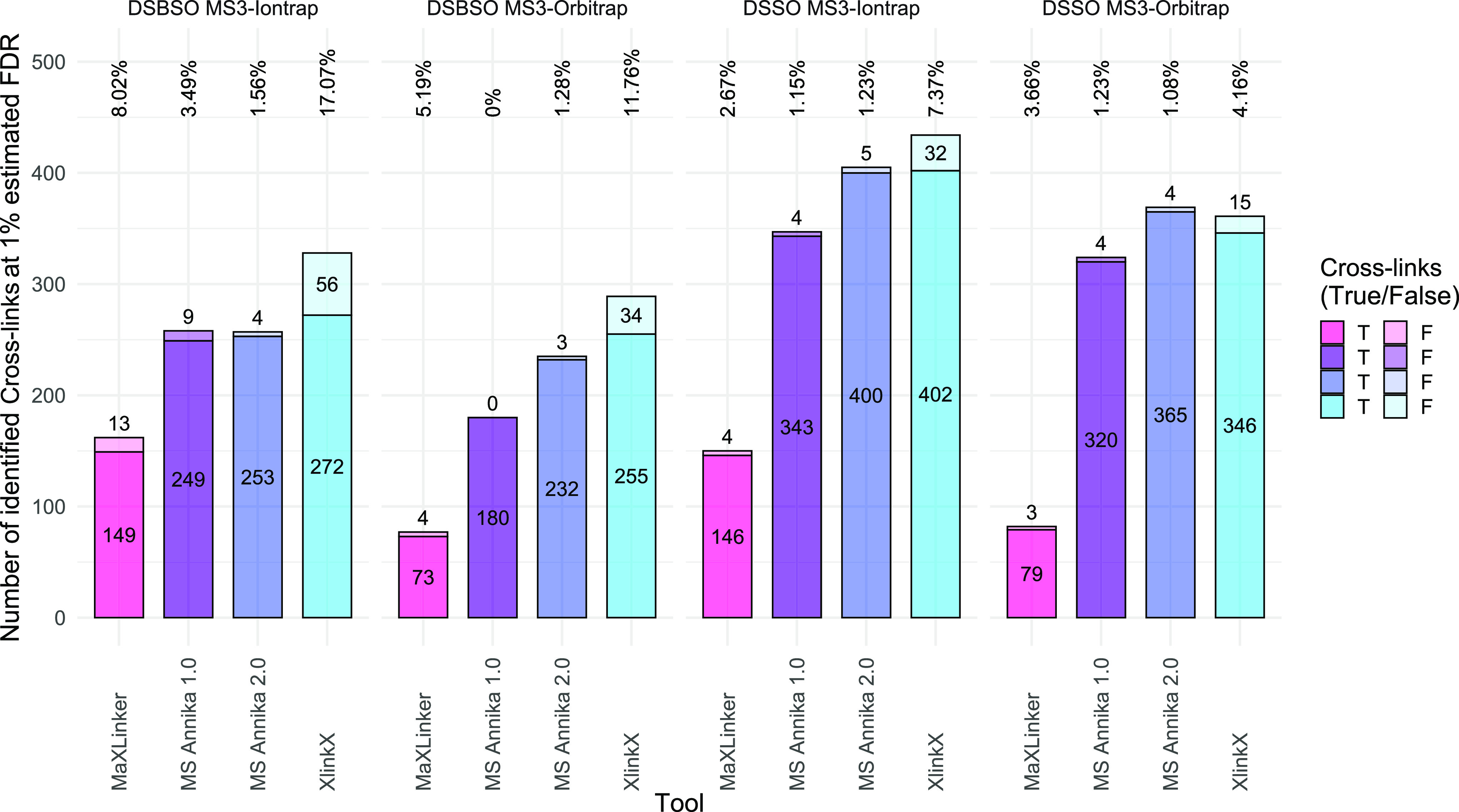
Results of the different cross-linking search engines at an estimated 1% FDR for dataset A. The figure is split into subplots for the four experiments, which are from left to right: the synthetic peptide library cross-linked with DSBSO and MS3 recorded in the ion trap, the library cross-linked with DSBSO and MS3 recorded in the Orbitrap, the library cross-linked with DSSO and MS3 recorded in the ion trap, and the library cross-linked with DSSO and MS3 recorded in the Orbitrap. True cross-links are colored in the darker shade and false cross-links in the lighter shade. Numbers above the bars denote the experimentally validated FDR. MS Annika 2.0 yields more true cross-links and better FDR estimates than MaXLinker in all cases, reporting between 104 to 286 more cross-links across the four datasets. Compared to XlinkX, MS Annika 2.0 finds less cross-links in 3 of the 4 datasets but provides better FDR estimates in all cases, with values ranging between 1.08 and 1.56% for MS Annika 2.0 and 4.16% up to 17.07% for XlinkX.

Using the synthetic peptide library datasets (datasets A, B, and C) allows us to calculate the experimentally validated FDR of our results and compare it to the estimated FDR of our search engine. The results for dataset A are depicted in Figure 2, which shows the performance of the MS2–MS3-based search approach of MS Annika 2.0 in comparison to the other cross-linking search engines MaXLinker and XlinkX at 1% estimated FDR. Additionally, we also compared this new search approach to the MS2-only search approach of the MS Annika version that we previously published^38^ (from hereon denoted as MS Annika 1.0). Figure 2 splits into four subplots for the different cross-linkers and mass analyzers. In all cases, MS Annika 2.0 yields true FDRs that are very close to the estimated FDR of 1% while still reporting high numbers of correctly identified cross-links. MS Annika 2.0 outperforms MS Annika 1.0 in terms of identified cross-links in all cases, as expected since the 1.0 version does not make use of the MS3 data. MS Annika 2.0 also yields more true cross-links and better experimentally validated FDRs than MaXLinker for all four of the different experiments, identifying between 104 and 286 more cross-links than MaXLinker at an experimentally validated FDR between 1.08 and 1.56%. For MaXLinker, the experimentally validated FDR ranges from 2.67 to 8.02%, noticeably worse than that of MS Annika 2.0. In comparison to XlinkX, MS Annika 2.0 identifies less true cross-links in three of the four cases but provides better FDR estimates in all cases as the experimentally validated FDR for XlinkX ranges between 4.16% and up to 17.07%. However, a direct comparison of the number of true cross-link identifications may not be suitable for cases where the experimentally validated FDR differs by a lot, for example, in the DSBSO ion trap dataset, where MS Annika 2.0 reports 4 false positive cross-links while XlinkX reports 56. We therefore also looked at the number of cross-link identifications at an experimentally validated 1% FDR (removing cross-links with the lowest score until less than 1% are false positives) and could confirm that MS Annika 2.0 provides the highest number of true cross-link identifications at an experimentally validated 1% FDR for all four datasets, outperforming MS Annika 1.0, MaXLinker, and XlinkX (see Figures S4–S7). Figure S8 shows the overlaps of the identified true cross-links of the different search engines. Generally, there is good agreement between MS Annika 2.0, MaXLinker, and XlinkX, except for the DSSO Orbitrap dataset, where XlinkX only has small overlaps with both MS Annika 2.0 and MaXLinker.

Interestingly, independently of the used search engine, more cross-links were identified in workflows using ion trap MS3 acquisition compared to workflows using Orbitrap MS3 acquisition, even though the Orbitrap yields higher resolution spectra. This can be attributed to the Orbitrap having longer cycle times and therefore a reduced scan rate, missing out on cross-linked peptides that cannot be sampled fast enough. The ion trap records spectra at a much lower resolution, but its speed and therefore ability to sample more cross-linked peptides make up for the reduced confidence in identifications that is caused by the lower resolution.

For dataset B,^26^ which is using the same synthetic peptide library as dataset A, the results are shown in Figure S9. For this dataset, the library was cross-linked with DSSO, and the MS3 was recorded in the Orbitrap, similar to one of the experiments of dataset A that we show in Figure 2. The results are also in line with what we could show for the experiment of dataset A: MS Annika 2.0 outperforms all other tools in terms of the number of true cross-link identifications, reporting 339 more cross-links than MaXLinker and 6 more than XlinkX, on average across the 3 replicates. MS Annika 2.0 simultaneously yields better FDR estimates than MaXLinker and XlinkX, providing an experimentally validated FDR of 4.09% on average, compared to 4.23% for MaXLinker and 10.15% for XlinkX. We also looked at overlaps in cross-link identification between the three replicates and the different search tools, which we show in the Venn diagrams depicted in Figure S10. The Venn diagrams show good agreement across replicates and between the search engines.

**Figure 3:**
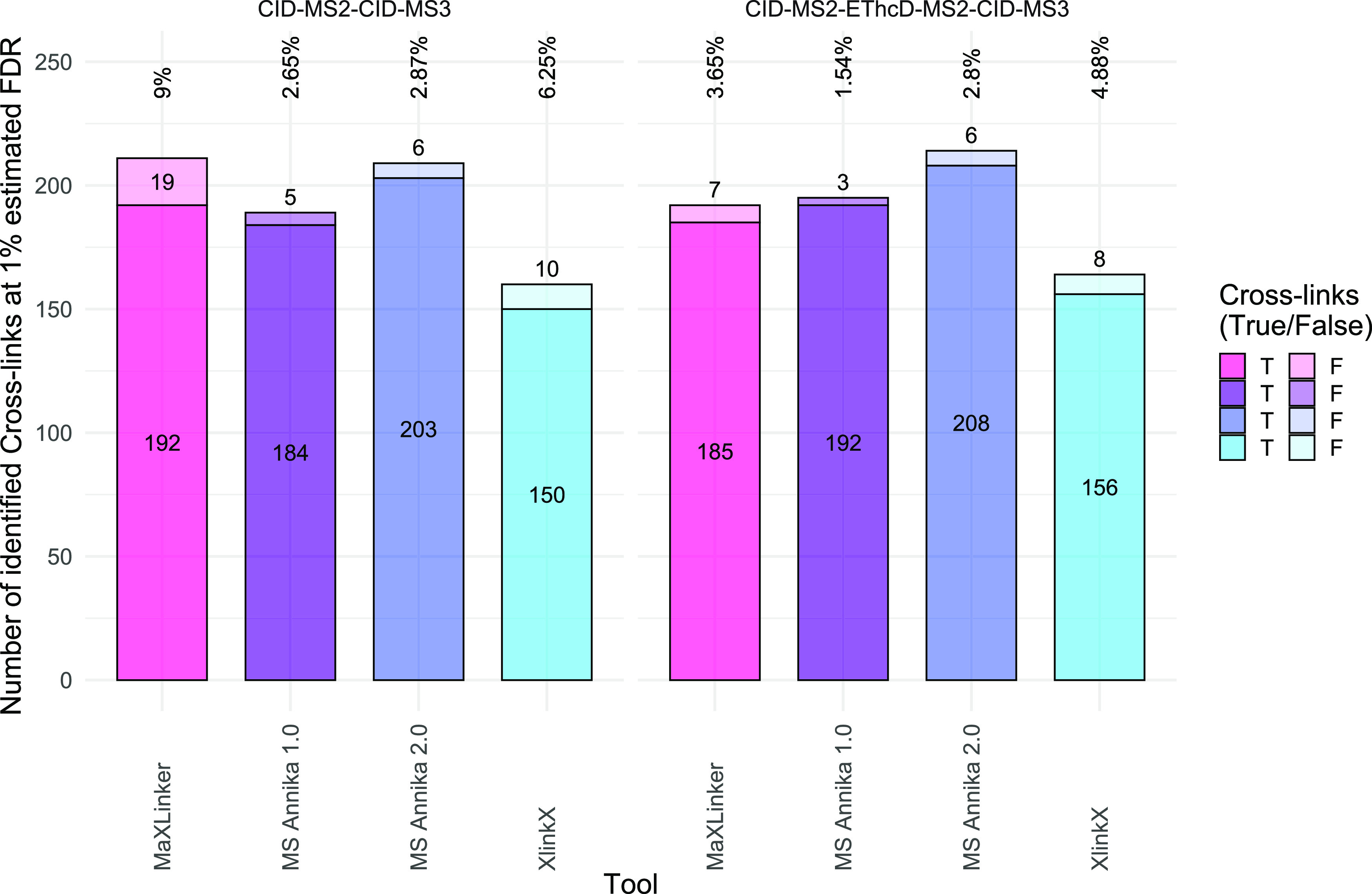
Overview of the results of the different search engines for dataset C.^13^ The figure is split into two subplots for the two different acquisition methods, CID-MS2-CID-MS3 on the left and CID-MS2-EThcD-MS2-CID-MS3 on the right. True cross-links are again colored in the darker shade and false positives in the lighter shade, while the numbers at the top denote the experimentally validated FDR. MS Annika 2.0 finds up to 23 more unique true cross-links than MaXLinker and up to 53 more than XlinkX, while also providing better FDR estimates for both acquisition methods. The experimentally validated FDR for MS Annika 2.0 is 2.87 and 2.80% for the two datasets, respectively, closer to the estimated 1% FDR target than MaXLinker and XlinkX, which yield experimentally validated FDRs of up to 9.00 and 6.25%, respectively. Numbers for XlinkX are taken from the publication of Beveridge and co-workers^13^ that we could also reproduce (±2 cross-links).

For dataset C, which is the synthetic peptide library of Beveridge and co-workers,^13^ MS Annika 2.0 exceeds MS Annika 1.0, MaXLinker, and XlinkX in the number of identified true cross-links in both the CID-MS2-CID-MS3 experiment and the CID-MS2-EThcD-MS2-CID-MS3 experiment, as shown in Figure 3. MS Annika 2.0 identifies up to 23 more cross-links than MaXLinker and up to 53 more cross-links than XlinkX. Moreover, MS Annika 2.0 also yields better FDR estimates than MaXLinker and XlinkX for both datasets, yielding experimentally validated FDRs of 2.87 and 2.80%, respectively. In comparison, MaXLinker provides experimentally validated FDRs of up to 9.00% and XlinkX up to 6.25%. These were also the only two datasets where we employed a score cut-off of 45 for XlinkX, as recommended by Beveridge and co-workers,^13^ which should improve FDR. Noticeably, MS Annika 2.0 still yields more true cross-links and better FDR estimates than XlinkX with the employed score cut-off. Omitting the score cut-off for XlinkX leads to slightly higher numbers of unique cross-link identifications of 165 and 161 true cross-links for the two datasets, respectively, but at the cost of much worse experimentally validated FDRs going up to 21.10 and 16.60%, respectively. In Figure S11, we again show good agreement between MS Annika 2.0, MaXLinker, and XlinkX in terms of identified true cross-links.

### MS Annika 2.0 Is the Cross-Linking Search Engine of Choice for Stepped HCD MS2 + CID MS3 Data

**Figure 4:**
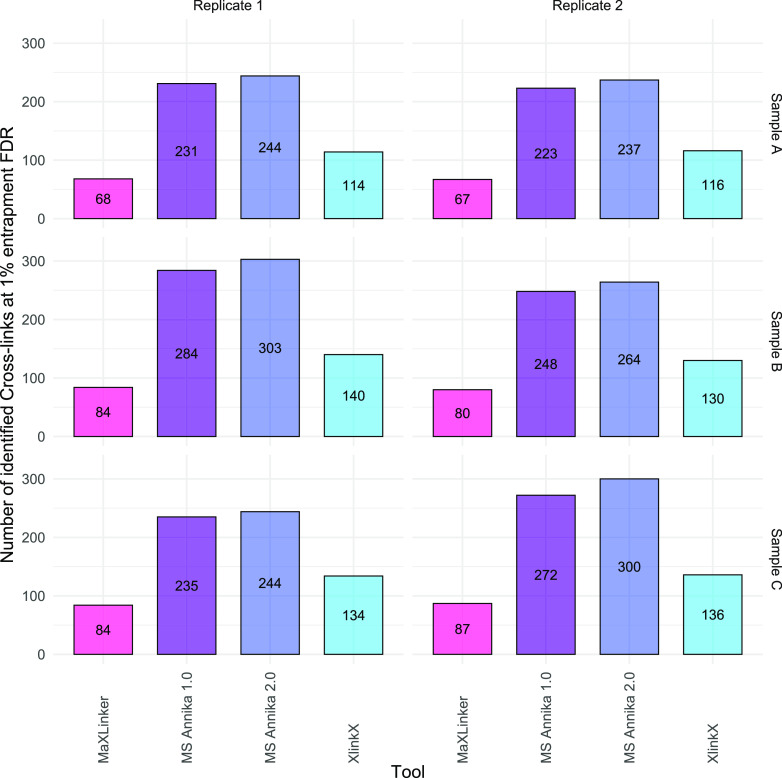
Number of identified cross-links at 1% entrapment FDR per search engine in dataset D by Ruwolt and co-workers^22^ who used stepped HCD MS2 acquisition in combination with CID MS3 scans. Samples A, B, and C are early, intermediate, and late SCX fractions, respectively, and the samples were analyzed in technical duplicates. MS Annika 2.0 outperforms all other search engines and identifies up to three times as many cross-links as MaXLinker and up to two times as many cross-links as XlinkX for all fractions and replicates.

The datasets by Ruwolt and co-workers^22^ (dataset D) featured spectra from a novel workflow design that uses stepped HCD for MS2 acquisition while additionally recording MS3 scans for cross-linked peptides using CID fragmentation. We analyzed these datasets using the human protein database they provided via PRIDE in combination with sequences from the *E. coli* proteome for better FDR control. Search results of MS Annika, MaXLinker, and XlinkX that were validated for 1% estimated FDR were post-processed as described in Section Data Processing and Evaluation by filtering out the lowest scoring cross-link until less than 1% of identified cross-links contained an *E. coli* peptide (which we know are false-positive hits as there was no *E. coli* in the sample). The post-processed results are summarized in Figure 4 for all SCX fractions and replicates. MS Annika 2.0 outperforms all other tools and yields approximately twice as many cross-links as XlinkX and more than three times as many as MaXLinker in all cases. More specifically, MS Annika 2.0 identifies 244 and 237 cross-links, respectively, in the two replicates of the early SCX fraction (Sample A). In comparison, MS Annika 1.0 identifies 231 and 223 cross-links, approximately 14 less than MS Annika 2.0. MaXLinker detects the lowest number of cross-links at 68 and 67 identifications, respectively, finding roughly 173 cross-links less than MS Annika 2.0. XlinkX does slightly better than MaXLinker at 114 and 116 cross-link identifications, respectively, but is still behind by about 126 cross-links compared to MS Annika 2.0. For the intermediate SCX fraction (sample B), MS Annika 2.0 provides 303 and 264 cross-links for the two replicates, respectively, again exceeding MS Annika 1.0, MaXLinker, and XlinkX. On average, MS Annika 2.0 identifies 18 more cross-links than MS Annika 1.0, 202 more cross-links than MaXLinker, and 149 more cross-links than XlinkX for sample B. In the late SCX fraction (sample C), MS Annika 2.0 again yields the highest number of cross-link identifications at 244 and 300 identifications for the two replicates, respectively. MS Annika 2.0 identifies 19 cross-links more than MS Annika 1.0, 187 cross-links more than MaXLinker, and 137 cross-links more than XlinkX on average for this SCX fraction. MS Annika 1.0 trails MS Annika 2.0 with anywhere from 9 to 28 less cross-links across all fractions and replicates, indicating that most of the cross-links can be identified in the MS2 spectra. Stepped HCD fragmentation has established itself as the method of choice for XLMS as the resulting spectra allow reliable identification of both cross-linked peptides.^20^ We can also observe this in our results, as the number of additional identifications, that are only found in MS3 spectra is considerably small. Figure S12 shows the overlaps of cross-link identifications across the replicates and search tools. Identifications are generally in good agreement across the different search engines, however, there is a noticeable variance between the replicates.

### MS Annika 2.0 Reveals Cross-Linked Residues Undetected by Other Cross-Linking Search Engines

**Figure 5:**
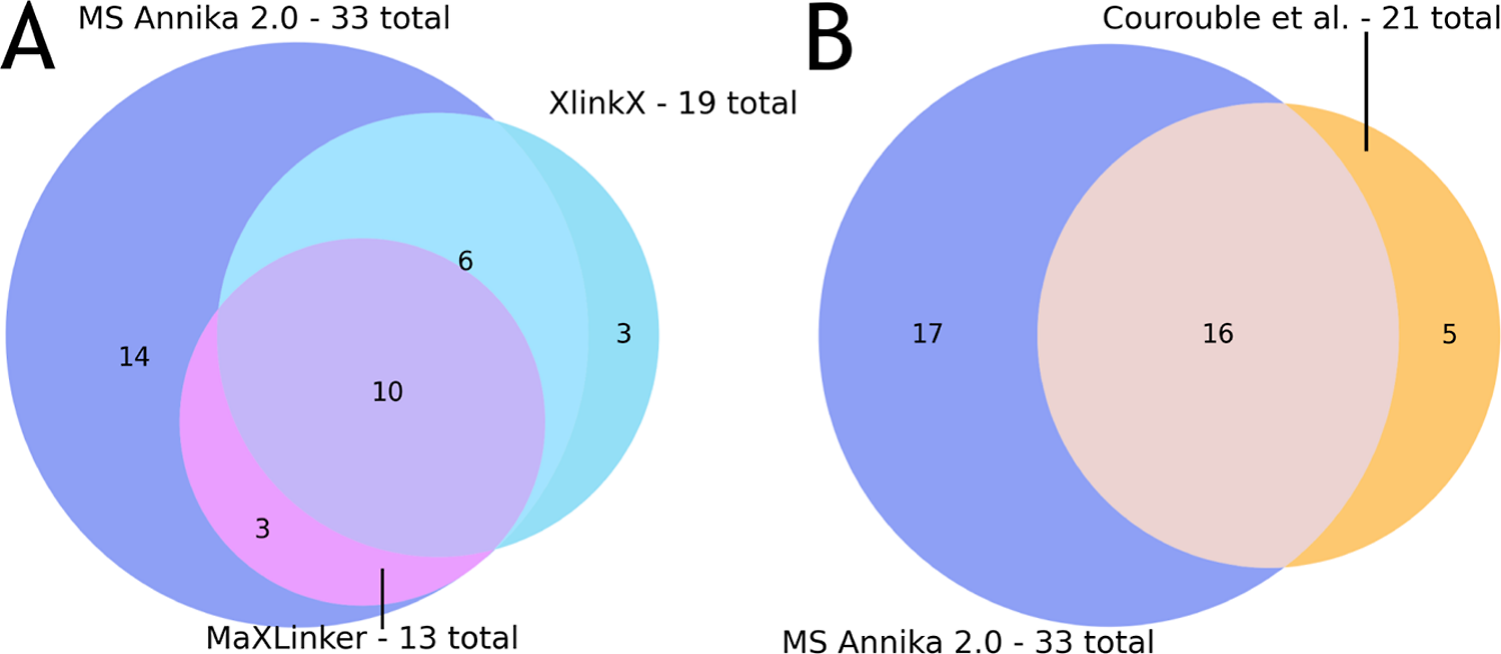
(A) For the NSP8 datasets by Courouble and co-workers (dataset E)^41^ MS Annika 2.0 outperforms all other search engines in terms of unique cross-link identifications at 33 cross-links found in total at a 1% estimated FDR. MaXLinker and XlinkX identify 13 and 19 unique cross-links, respectively. Remarkably, MS Annika 2.0 detects all 13 cross-links identified by MaXLinker and 16 of the 19 cross-links identified by XlinkX, while additionally finding 14 more cross-links that are not detected by either MaXLinker or XlinkX. (B) Comparison of crosslinks detected by MS Annika 2.0 to the cross-links reported in the publication by Courouble and co-workers,^41^ who also used XlinkX for cross-link identification. MS Annika 2.0 could identify 16 of the 21 cross-links they reported while finding 17 additional, not yet described cross-links on top of that.

In the cross-linking experiment by Courouble and co-workers,^41^ that we re-analyzed, NSP8 was cross-linked with DSSO to uncover the structural plasticity of the protein. In Figure 5A, we show the number of unique cross-links identified by MS Annika 2.0, MaXLinker, and XlinkX at 1% estimated FDR across the three replicates and the overlap of the results between the three search engines. MS Annika 2.0 yields the highest number of cross-link identifications at 33 unique cross-links, while MaXLinker identifies 13 and XlinkX identifies 19 unique cross-links in total. All cross-links detected by MaXLinker are also found by MS Annika 2.0, and of the 19 cross-links identified by XlinkX, 16 are also detected by MS Annika 2.0. Out of the 33 cross-links identified by MS Annika 2.0, 19 are also detected by either MaXLinker or XlinkX, leaving 14 cross-links that are exclusively detected by MS Annika 2.0. We additionally compared our results to the cross-links reported in the publication by Courouble and co-workers, who also used XlinkX for cross-link identification. The comparison is depicted in Figure 5B, which shows that MS Annika 2.0 also finds 16 of the 21 cross-links they reported. Moreover, MS Annika 2.0 identifies 17 additional cross-links that are not reported in their publication. In order to validate these 17 cross-links, we mapped them to the 3D structure of the NSP7-NSP8-complex that we retrieved from the protein data bank^55^ (PDB) using the identifier 6YHU,^56^ which was also used by Courouble and co-workers in their publication. The 3D structure including the mapped cross-links is shown in Supplementary Figure S13. Out of the 17 additional cross-links found by MS Annika 2.0, we could map four to the 3D structure, the other 13 correspond to peptide sequences that are not resolved in this PDB complex. Since the NSP7-NSP8 complex is a multimer consisting of two NSP7 and two NSP8, three of the four cross-links are ambiguous as the cross-linked peptides appear in two different chains, thereby creating four pairs of cross-linked residues per cross-link. As shown in Figure S13, the four mappable cross-links found by MS Annika 2.0 result in 13 pairs of cross-linked residues when they are mapped to this structure. Even though the spacer arm of DSSO is only 10.1 Å long,^17^ the maximum Cα – Cα distance for DSSO cross-linked residues is reported to be between 27 and 35 Å^34,57^ due to the flexibility of lysine side chains and backbone dynamics. We used the more conservative distance threshold of 27 Å for validating the cross-links identified by MS Annika 2.0. Most importantly, for each of the four cross-links, we could find a corresponding cross-linked residue pair that falls within this distance constraint of 27 Å. This validates that at least the four mappable cross-links are indeed true identifications by MS Annika 2.0.

### MS Annika 2.0 Results Can be Exported to a Variety of Different Visualization Tools

In addition to the exporting options offered by Proteome Discoverer, MS Annika 2.0 results can now be exported to a variety of cross-link visualization tools commonly used in the field. Foremost, we developed an exporter for the PyMOL plugin PyXlinkViewer,^50^ which facilitates the visualization of cross-links in a 3D protein structure and colors cross-links based on their length, allowing easy assessment if a cross-link satisfies the cross-linker-specific distance constraint. Similar results are possible in ChimeraX^53^ with the plugin XMAS^54^ which supports MS Annika 2.0 result files out-of-the-box and is able to generate publication ready figures as shown in Figure S13. Furthermore, we implemented exporters to xiNET^48^ and xiVIEW,^49^ tools that aid in the downstream analysis and interpretation of cross-link identifications. Exemplary usage of the MS Annika exporters is shown in the Supporting Information.

## Discussion and Conclusions

We have implemented a novel MS2–MS3 search algorithm in our cross-linking search engine, MS Annika 2.0, that allows the analysis of data originating from MS2–MS3-based cross-linking workflows. Our algorithm utilizes MS2 and MS3 spectra to derive accurate precursor masses that allow reliable identification of the cross-linked peptides. Furthermore, we developed a new scoring function that boosts peptides identified across multiple tandem MS stages. We benchmarked our algorithm against MaXLinker and XlinkX, two other search engines commonly used for cross-link identification in MS2–MS3-based cross-linking experiments. For this benchmarking process, we used datasets from five different cross-linking experiments of independent labs covering a variety of cross-linkers, workflow designs, instruments, acquisition methods, and fragmentation methods, as well as different use cases. We could show that our MS2–MS3 search algorithm identifies high numbers of cross-links in both low- and high-resolution MS3 spectra across all datasets, outperforming MaXLinker and XlinkX in almost all of the cases. Three of the experiments were analyzing synthetic peptide libraries that allow the calculation of an experimentally validated FDR, and we could show that our MS2–MS3 search algorithm yields more accurate FDR estimates than both MaXLinker and XlinkX for every dataset. Additionally, we have shown that our search algorithm is able to uncover cross-linked residues that are not detected by any of the other search tools, providing further insight into the structural and functional relationships of the underlying proteins.

Several publications have shown that stepped HCD MS2 acquisition can outperform current MS2–MS3-based approaches in terms of the number of identifiable cross-links.^13,20−22,26^ Ruwolt and co-workers supplemented stepped HCD MS2 acquisition with additional MS3 scans for detected cross-link doublets, which performed better than traditional CID-MS2-CID/HCD-MS3 acquisition but ultimately still fell short to the standard stepped HCD MS2 approach due to the longer duty cycles,^22^ however, only by a small margin (as seen in Figure S14). Even though stepped HCD acquisition potentially yields more unique cross-link identifications, MS2–MS3 workflows are still widely used in the cross-linking community for several reasons: first, MS2–MS3 acquisition outperforms regular MS2 and MS2–MS2 acquisition and is therefore the method of choice when stepped HCD is not an option. Second, MS2–MS3 acquisition provides higher sequence coverage^21^ and more accurate quantification results^22^ than stepped HCD workflows. Third, stepped HCD acquisition results are highly dependent on the used collision energies,^20^ making it necessary to tune acquisition parameters to optimize performance. On the other hand, MS2–MS3 acquisition is well established and uses fixed collision energies of usually 25% for MS2 acquisition and 35% for MS3 acquisition, with very little need for manual tuning. Moreover, continuous research is conducted aiming to improve MS3 acquisition, for example, Kolbowski and co-workers recently proposed a novel algorithm for MS3 decision-making that could improve which peptides are selected for MS3 acquisition.^58^ Considering the ongoing developments in MS3 acquisition, we believe that MS2–MS3-based workflows have the potential to overtake stepped HCD MS2 acquisition in the future. With our updated MS Annika 2.0 algorithm capable of analyzing MS3 data, we present a tool that makes the most of the available information, reliably detecting high numbers of cross-links at accurate FDR estimates. We are convinced that the results will improve further with the continuing advancements in mass spectrometry instruments and MS3 acquisition.

## Availability and Limitations

The new MS2–MS3 search algorithm is implemented in C# in our existing cross-linking search engine MS Annika that is available as node for Proteome Discoverer. The updated version—MS Annika 2.0—including the MS2–MS3 search is available free of charge for Proteome Discoverer 2.5, 3.0, and 3.1 at https://ms.imp.ac.at/index.php?action=ms-annika or via the GitHub repository https://github.com/hgb-bin-proteomics/MSAnnika. The MS Annika 2.0 plugin node can be run with a free version of Proteome Discoverer, which you can download from the Thermo Fisher Web site https://www.thermofisher.com/at/en/home/industrial/mass-spectrometry/liquid-chromatography-mass-spectrometrylc-ms/lc-ms-software/multi-omics-data-analysis/proteomediscoverer-software.html. MS Annika 2.0 makes use of the graphical user interface for workflow generation in Proteome Discoverer so users can easily set up searches with minimal bioinformatics knowledge. The MS2–MS3 search algorithm uses the isotope patterns in the MS2 spectra at several steps, it is therefore important to not deisotope the MS2 spectra before cross-link search. Furthermore, since MS Annika 2.0 uses MSn spectra, it needs MSn stage as well as MS2- and MS3-precursor information, which is why MGF files are not supported. A detailed user manual, including descriptions of all tunable parameters and step-by-step instructions for running MS Annika 2.0, as well as license information is also given on the MS Annika 2.0 webpage. Pre- and post-processing scripts have been published via the GitHub repositories https://github.com/hgb-bin-proteomics/MaXLinker_extensions and https://github.com/hgb-bin-proteomics/MSAnnika_exporters. All mass spectrometry proteomics data along with result files have been deposited to the ProteomeXchange consortium (http://proteomecentral.proteomexchange.org) via the PRIDE partner repository^42^ with the dataset identifier PXD041955. Result files including scripts for analysis and visualization are also available via GitHub at https://github.com/hgb-bin-proteomics/MSAnnika_MS3_Results.

## Supporting Information Available

Cross-link search workflow design and search parameters, MS Annika exporter script usage, dataset specific search parameters, Proteome Discoverer workflow used for cross-link identification, typical MS2–MS3 cross-linking workflow, fragmentation behavior of DSSO, dataset A DSBSO-IT: cross-links identified at 1 and 5% experimentally validated FDR, dataset A DSBSO-OT: cross-links identified at 1 and 5% experimentally validated FDR, dataset A DSSO-IT: cross-links identified at 1 and 5% experimentally validated FDR, dataset A DSSO-OT: cross-links identified at 1 and 5% experimentally validated FDR, dataset A: overlaps of correctly identified cross-links, dataset B: number of identified cross-links and experimentally validated FDR of the different search engines at 1% estimated FDR, dataset B: overlaps of correctly identified cross-links, dataset C: overlaps of correctly identified cross-links, dataset D: overlaps of correctly identified cross-links, dataset E: crosslinks uniquely identified by MS3 Annika mapped to the 3D structure of the NSP7-NSP8 complex, and dataset D: comparison of stepped HCD versus stepped HCD + MS3 acquisition (PDF)
