# Supplementary material for: MS Annika 2.0 Identifies Cross-Linked Peptides in MS2–MS3-Based Workflows at High Sensitivity and Specificity
Source: J Proteome Res. 2023 Aug 11;22(9):3009–21. doi: 10.1021/acs.jproteome.3c00325 (PMC10476269; doi:10.1021/acs.jproteome.3c00325)
Supplement: Supplementary file 1 — pr3c00325_si_001.pdf [file pr3c00325_si_001.pdf]

MS Annika 2.0 identifies cross-linked peptides in MS2-MS3-based workflows at high sensitivity and specificity - Supplementary Material

Micha Johannes Birklbauer*,[1], Manuel Matzinger[2], Fränze Müller[2], Karl Mechtler[2,3,4], Viktoria Dorfer*,[1]

[1] University of Applied Sciences Upper Austria, Bioinformatics Research Group, Softwarepark 11, 4232 Hagenberg, Austria

[2] Institute of Molecular Pathology (IMP), Vienna BioCenter (VBC), Campus-Vienna-Biocenter 1, 1030 Vienna, Austria

[3] Institute of Molecular Biotechnology (IMBA), Austrian Academy of Sciences, Vienna BioCenter (VBC), Dr. Bohr-Gasse 3, 1030 Vienna, Austria

[4] Gregor Mendel Institute (GMI), Austrian Academy of Sciences, Vienna BioCenter (VBC), Dr. Bohr-Gasse 3, 1030 Vienna, Austria

*E-mail: micha.birklbauer@fh-hagenberg.at, viktoria.dorfer@fh-hagenberg.at

Table of Contents

Table of Contents S1
Crosslink search workflow design and search parameters S2
MS Annika exporter script usage S3
Supplementary Table S1 S4
Supplementary Figure S1 S5
Supplementary Figure S2 S6
Supplementary Figure S3 S7
Supplementary Figure S4 S8
Supplementary Figure S5 S9
Supplementary Figure S6 S10
Supplementary Figure S7 S11
Supplementary Figure S8 S12
Supplementary Figure S9 S13
Supplementary Figure S10 S14
Supplementary Figure S11 S15
Supplementary Figure S12 S16
Supplementary Figure S13 S17
Supplementary Figure S14 S18
References S19

Crosslink search workflow design and search parameters

Global parameters used across all search engines and all datasets:

- Max. Missed Cleavages: 4
- Fixed Modification: Carbamidomethyl (C) +57.021 Da
- Variable Modification: Oxidation (M) +15.995 Da
- Monolink Modifications for DSSO:
  - DSSO Amidated (K) +175.030 Da
  - DSSO Hydrolysed (K) +176.014 Da
  - DSSO Tris (K) +279.078 Da
- Monolink Modifications for DSBSO:
  - DSBSO Amidated (K) +325.065 Da
  - DSBSO Hydrolysed (K) +326.049 Da
  - DSBSO Tris (K) +429.113 Da

Global MS Annika 2.0 parameters used across all datasets:

- Minimum Charge: 0
- Additional Crosslink Doublet Distances: 49.982635 for DSSO, 200.0177 for DSBSO
- Top N most intense doublets: 3
- Multiplicative Penalty for Crosslinks with Equal Sequences: 1

A more detailed description of the specific parameters in MS Annika/MS Annika 2.0 is given in the MS Annika manual that can be found here: https://github.com/hgb-bin-proteomics/MSAnnika

For a more detailed overview of how the doublet detection and MS2 search works in MS Annika/MS Annika 2.0, please refer to the MS Annika 1.0 publication: https://doi.org/10.1021/acs.jproteome.0c01000

Supplementary Table S1 and ***Supplementary Figure S1*** show the exact parameters and workflow used for crosslink identification.

MS Annika exporter script usage

All of the scripts use Microsoft Excel files as input, for that MS Annika results (any MS Annika version works) need to be exported from Proteome Discoverer by opening the “crosslink” result table and then select “File > Export > To Microsoft Excel… > Level 1: Crosslinks > Export”

Requirements for running the scripts: python 3.7+, pandas, openpyxl, biopython, biopandas

Exporting to xiNET:

Files needed:

- result.xlsx - MS Annika result file(s) exported to .xlsx
- seq.fasta - FASTA file containing sequences of the crosslinked proteins

“python xiNetExporter_msannika.py result.xlsx -fasta seq.fasta”

Exporting to xiVIEW:

Files needed:

- result.xlsx - MS Annika result file(s) exported to .xlsx
- seq.fasta - FASTA file containing sequences of the crosslinked proteins

“python xiViewExporter_msannika.py result.xlsx -fasta seq.fasta”

Exporting to pyXlinkViewer (pyMOL):

Files needed:

- result.xlsx - MS Annika result file(s) exported to .xlsx
- structure.pdb - 3D structure of the protein (complex) that crosslinks should be mapped to

“python pyXlinkViewerExporter_msannika.py result.xlsx -pdb structure.pdb”

Scripts and documentation are available at
https://github.com/hgb-bin-proteomics/MSAnnika_exporters

Supplementary Table S1

- Dataset A
  Tolerances denoted with a slash are orbitrap tolerance/ion trap tolerance

	MS Annika 2.0	MaXLinker	XlinkX
**MS1 Tolerance**	5 ppm	5 ppm	5 ppm
**MS2 Tolerance**	10 ppm	0.02 Da	10 ppm
**MS3 Tolerance**	10 ppm/0.5 Da	0.02 Da/0.5 Da	10 ppm/0.5 Da

- Dataset B

	MS Annika 2.0	MaXLinker	XlinkX
**MS1 Tolerance**	5 ppm	5 ppm	5 ppm
**MS2 Tolerance**	10 ppm	0.02 Da	10 ppm
**MS3 Tolerance**	10 ppm	0.02 Da	10 ppm

- Dataset C

	MS Annika 2.0	MaXLinker	XlinkX
**MS1 Tolerance**	5 ppm	10 ppm	5 ppm
**MS2 Tolerance**	10 ppm	0.05 Da	10 ppm
**MS3 Tolerance**	0.5 Da	0.5 Da	0.5 Da

- Dataset D

	MS Annika 2.0	MaXLinker	XlinkX
**MS1 Tolerance**	5 ppm	5 ppm	5 ppm
**MS2 Tolerance**	10 ppm	0.02 Da	10 ppm
**MS3 Tolerance**	0.5 Da	0.5 Da	0.5 Da

- Dataset E

	MS Annika 2.0	MaXLinker	XlinkX
**MS1 Tolerance**	10 ppm	10 ppm	10 ppm
**MS2 Tolerance**	20 ppm	0.02 Da	20 ppm
**MS3 Tolerance**	0.5 Da	0.5 Da	0.5 Da

Table S1: Dataset specific search parameters used across the different search engines.

Supplementary Figure S1

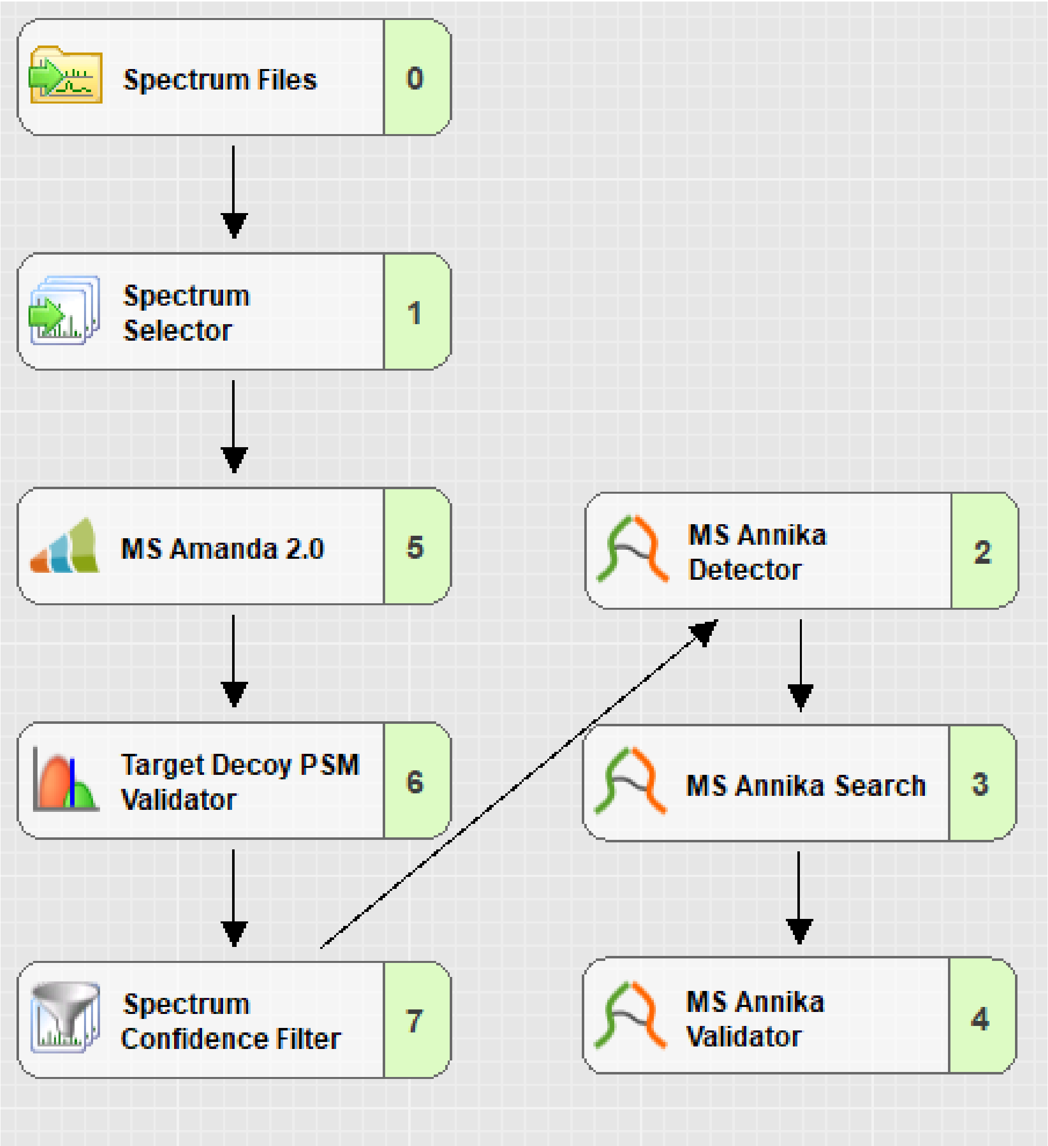

Fig. S1: The above screenshot shows the Proteome Discoverer workflow design used for identifying crosslinks with MS Annika 2.0. Spectra containing linear peptides and monolinks are first filtered out using MS Amanda and any remaining spectra are searched for crosslinks with the MS Annika 2.0 nodes.

Supplementary Figure S2

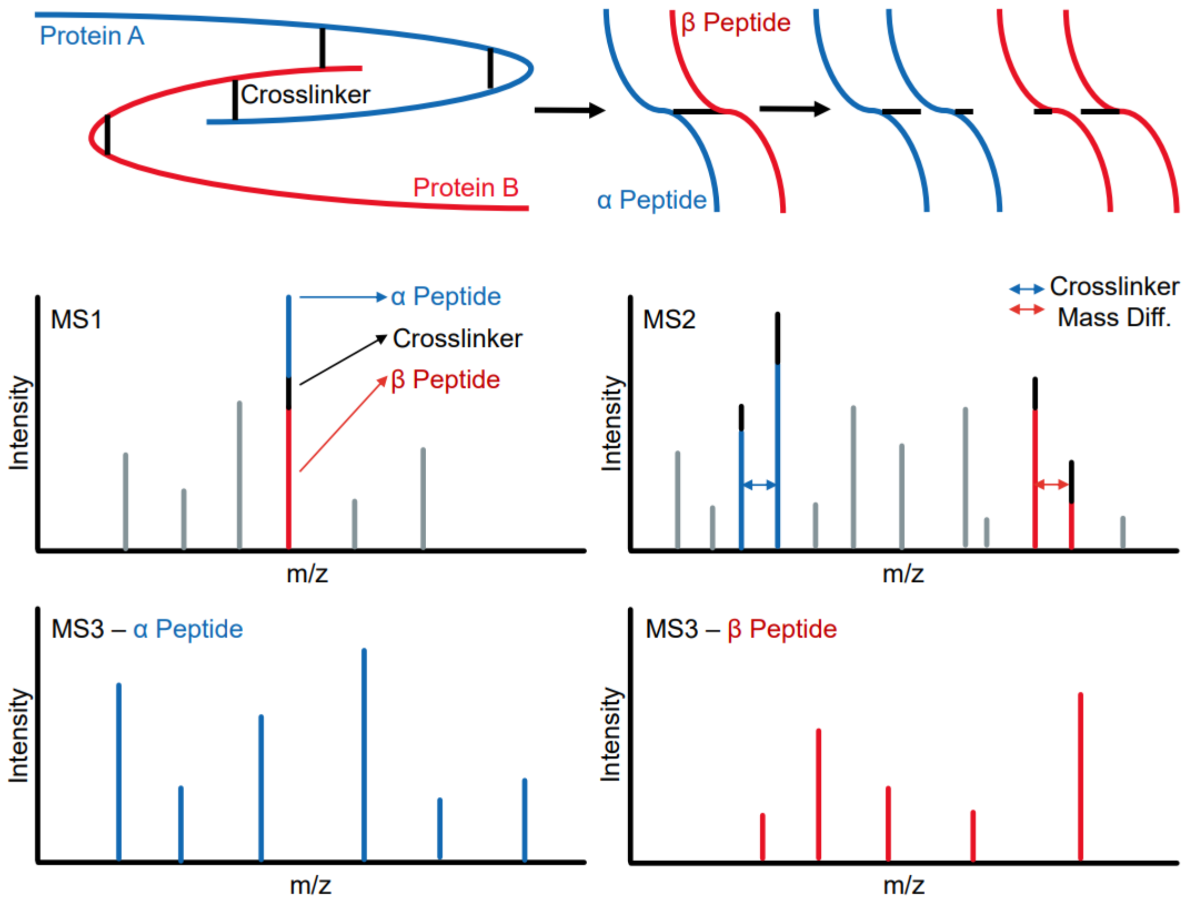

Fig. S2: A typical MS2-MS3 cross-linking workflow using a cleavable crosslinker. A crosslinker is added to the sample to covalently link residues in close spatial proximity forming crosslinks either within the same protein (intralink) or within different proteins (interlink). After digestion the cross-linked proteins are broken up into several cross-linked peptides that are analysed with LC-MS. In the MS1 scan the two peptides are still cross-linked together, yielding a peak of the whole cross-linked entity. For the MS2 scan, the cross-linked peptides are fragmented, the crosslinker is cleaved and the two peptides are broken up. Because the crosslinker incorporates an off-centre cleavage site each peptide is modified with either the shorter fragment or the longer fragment of the crosslinker, resulting in doublet peaks (coloured in blue and red in the MS2 scan) that show a crosslinker specific mass difference (e.g., 32 Da for DSSO). The doublet peaks are usually referred to by their crosslinker modification (mass) and the corresponding peptide, for example *alpha light* for the leftmost blue doublet peak: doublet peak originates from the *alpha* peptide and the respective crosslinker modification is the short fragment which yields a smaller mass, therefore it is the *light* peak (in comparison to the longer fragment that yields the *heavy* peak). For doublet peaks an MS3 scan is recorded after the MS2 scan where each MS3 scan should only contain one of the two cross-linked peptides in linear form.

Supplementary Figure S3

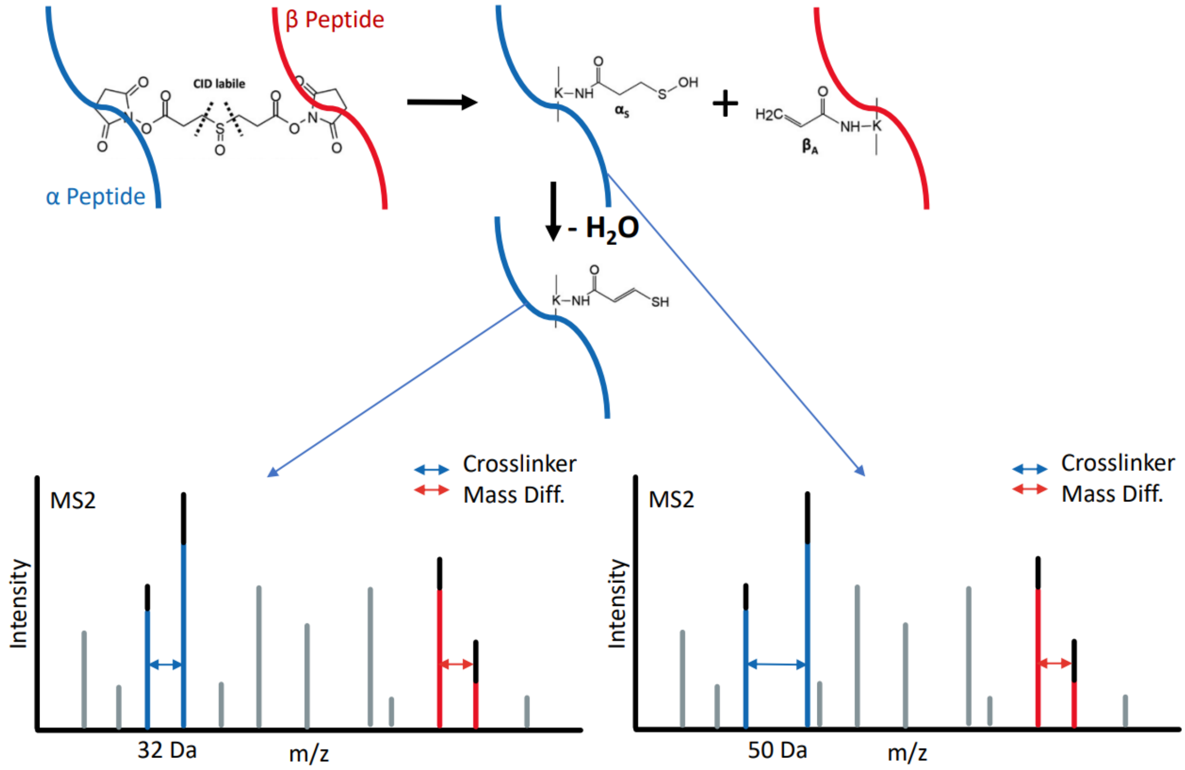

Fig. S3: Fragmentation behaviour of DSSO[1] (exemplary also for other sulfoxy-based crosslinkers like DSBSO). During fragmentation the two peptides cross-linked with DSSO (top left) are cleaved which results in the beta peptide carrying the alkene fragment (+54 Da) and the alpha peptide carrying the sulfenic acid fragment (+104 Da) (top right) and vice versa (not depicted). This pairing of a peptide modified with alkene and a peptide modified with sulfenic acid results in a doublet with a mass difference of 50 Da, as depicted in the MS2 scan in the bottom right. However, during fragmentation the sulfenic acid fragment might lose a water molecule, transforming it into the thiol fragment instead. This creates a pairing of peptide modified with the alkene fragment (+54 Da) and peptide modified with the thiol fragment (+86 Da), resulting in a different doublet with a mass difference of 32 Da, as depicted in the bottom left. Detection of both cases by the search engine is essential for the correct calculation of the unmodified peptide's mass which subsequently is important for peptide identification. Graphics of DSSO are taken from the publication by Kao et al[1].

Supplementary Figure S4

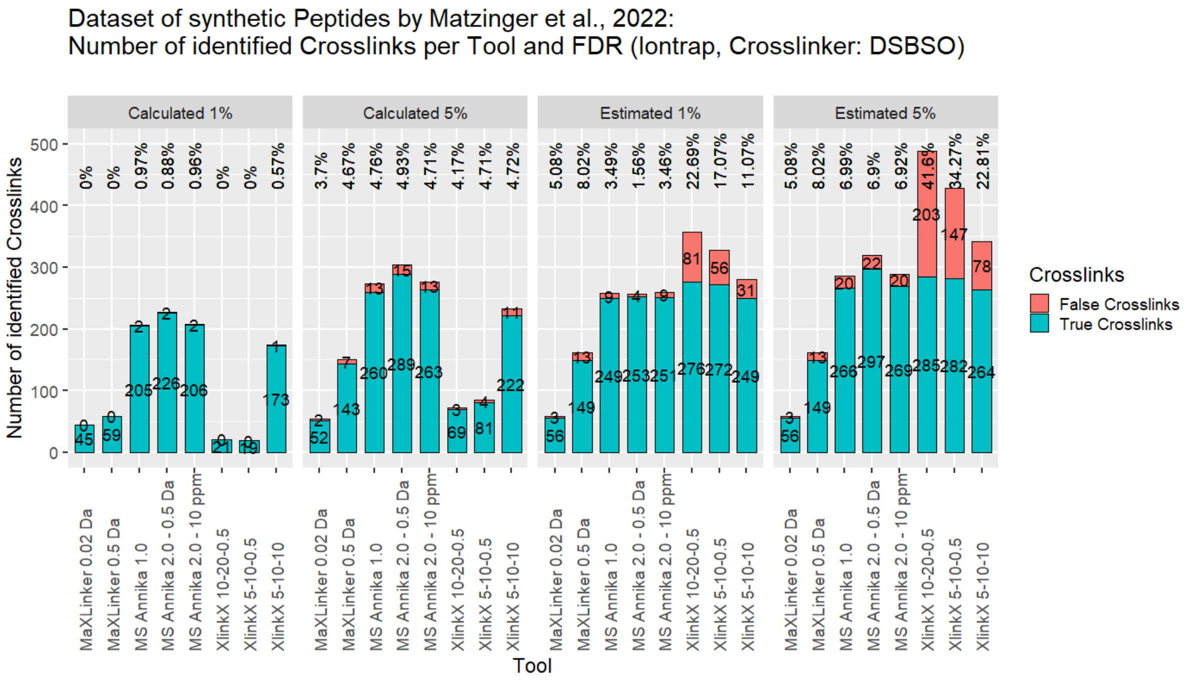

Fig. S4: *Dataset A (DSBSO Ion trap experiment)* - Number of crosslinks identified at 1% and 5% experimentally validated FDR (here denoted as “calculated FDR”) and at 1% and 5% estimated FDR for the different search engines and search settings. Numbers at the top denote the calculated, experimentally validated FDR. Numbers after the search engine name denote used tolerances, e.g., MaXLinker 0.02 Da denotes a 0.02 Da tolerance for MS3 scans and XlinkX 10-20-0.5 denotes MS1 and MS2 tolerances of 10/20 ppm and MS3 tolerance of 0.5 Da. MS Annika 2.0 outperforms all other tools at 1% and 5% experimentally validated FDR. At 1% experimentally validated FDR MS Annika 2.0 detects almost 4 times as many crosslinks as MaXLinker and 31% more than XlinkX. At 5% experimentally validated FDR MS Annika 2.0 provides twice as many crosslinks as MaXLinker and 30% more than XlinkX. MS Annika 2.0 also yields accurate FDR estimates, very close to the experimentally validated values for both 1% and 5% FDR results. XlinkX noticeably suffers from poor FDR estimation which is even further exaggerated by validating for a more relaxed 5% estimated FDR.

Supplementary Figure S5

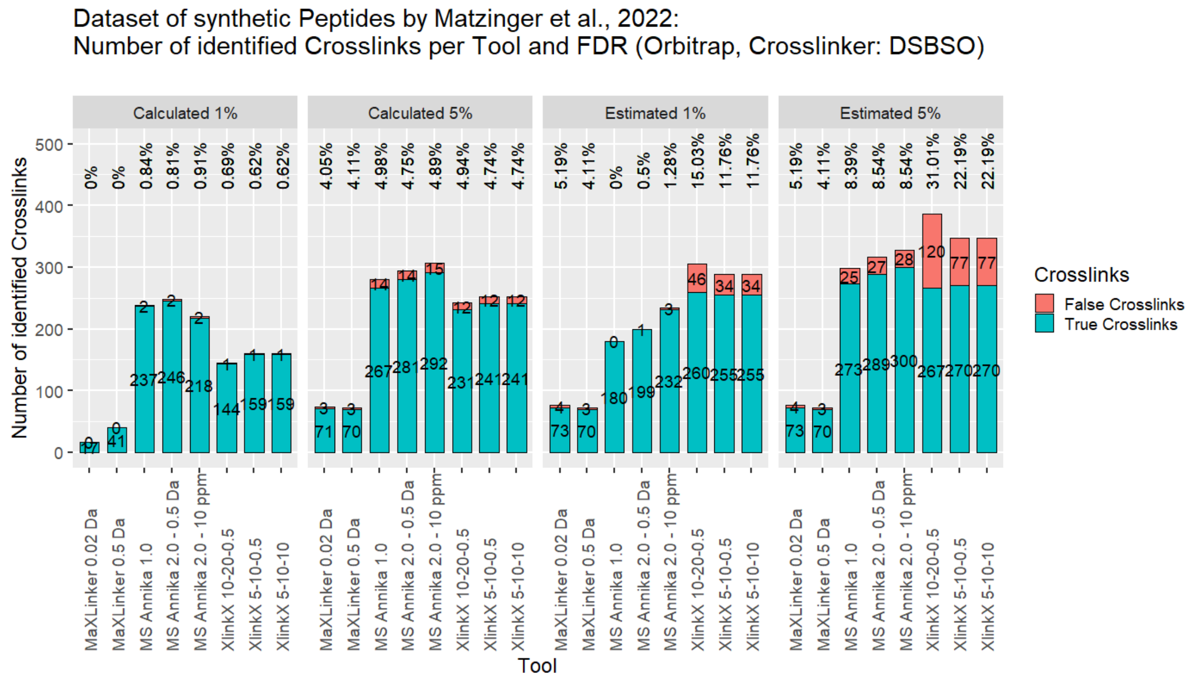

Fig. S5: *Dataset A (DSBSO Orbitrap experiment)* - Number of crosslinks identified at 1% and 5% experimentally validated FDR (here denoted as “calculated FDR”) and at 1% and 5% estimated FDR for the different search engines and search settings. Numbers at the top denote the calculated, experimentally validated FDR. Numbers after the search engine name denote used tolerances, e.g., MaXLinker 0.02 Da denotes a 0.02 Da tolerance for MS3 scans and XlinkX 10-20-0.5 denotes MS1 and MS2 tolerances of 10/20 ppm and MS3 tolerance of 0.5 Da. MS Annika 2.0 outperforms all other tools at 1% and 5% experimentally validated FDR. At 1% experimentally validated FDR MS Annika 2.0 detects almost 6 times as many crosslinks as MaXLinker and 55% more than XlinkX. At 5% experimentally validated FDR MS Annika 2.0 provides 4 times as many crosslinks as MaXLinker and 21% more than XlinkX. MS Annika 2.0 also yields accurate FDR estimates, very close to the experimentally validated values for both 1% and 5% FDR results. Again XlinkX noticeably suffers from poor FDR estimation which is even further exaggerated by validating for a more relaxed 5% estimated FDR.

Supplementary Figure S6

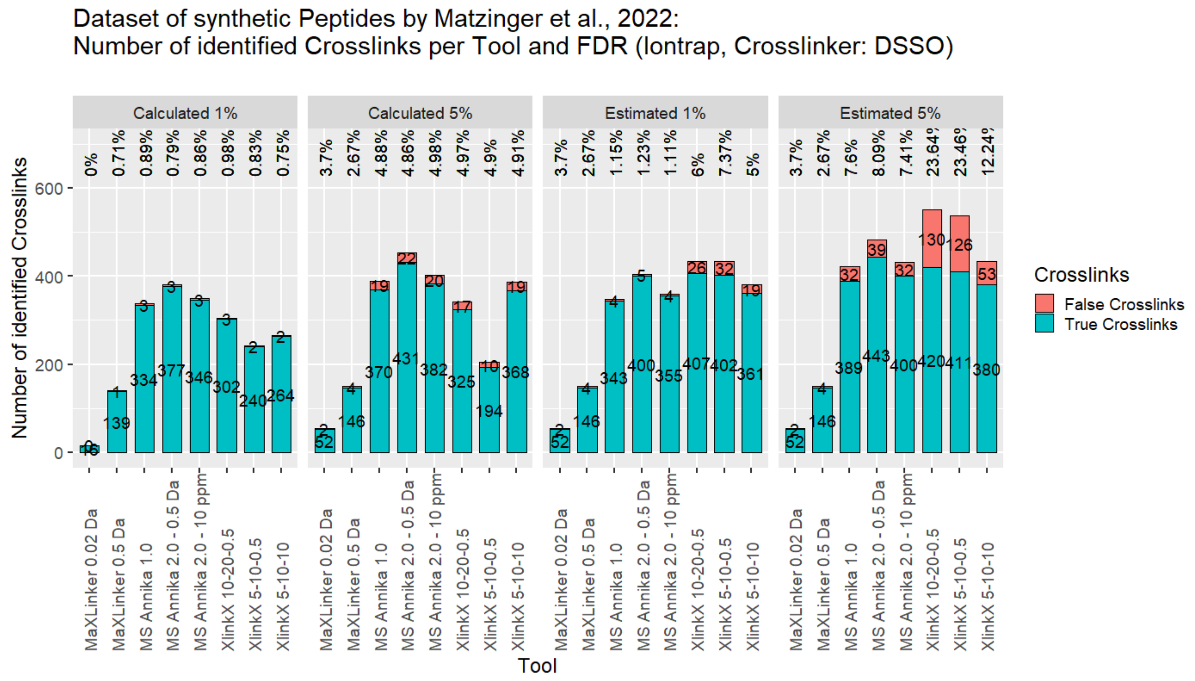

Fig. S6: *Dataset A (DSSO Ion trap experiment)* - Number of crosslinks identified at 1% and 5% experimentally validated FDR (here denoted as “calculated FDR”) and at 1% and 5% estimated FDR for the different search engines and search settings. Numbers at the top denote the calculated, experimentally validated FDR. Numbers after the search engine name denote used tolerances, e.g., MaXLinker 0.02 Da denotes a 0.02 Da tolerance for MS3 scans and XlinkX 10-20-0.5 denotes MS1 and MS2 tolerances of 10/20 ppm and MS3 tolerance of 0.5 Da. MS Annika 2.0 outperforms all other tools at 1% and 5% experimentally validated FDR. At 1% experimentally validated FDR MS Annika 2.0 detects almost three times as many crosslinks as MaXLinker and 25% more than XlinkX. At 5% experimentally validated FDR MS Annika 2.0 provides three times as many crosslinks as MaXLinker and 17% more than XlinkX. MS Annika 2.0 also yields accurate FDR estimates, very close to the experimentally validated values for both 1% and 5% FDR results. Again XlinkX noticeably suffers from poor FDR estimation, however it does slightly better for DSSO compared to DSBSO. The bad FDR estimation is further exaggerated by validating for a more relaxed 5% estimated FDR, increasing the experimentally validated FDR up to almost 24%.

Supplementary Figure S7

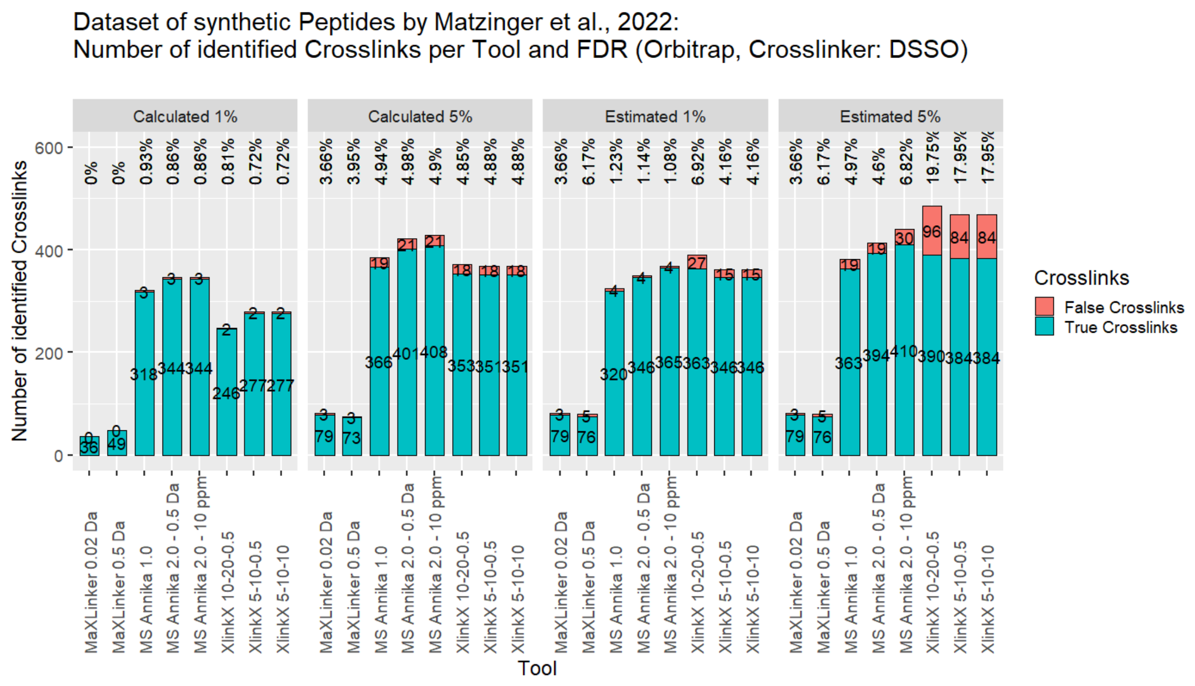

Fig. S7: *Dataset A (DSSO Orbitrap experiment)* - Number of crosslinks identified at 1% and 5% experimentally validated FDR (here denoted as “calculated FDR”) and at 1% and 5% estimated FDR for the different search engines and search settings. Numbers at the top denote the calculated, experimentally validated FDR. Numbers after the search engine name denote used tolerances, e.g., MaXLinker 0.02 Da denotes a 0.02 Da tolerance for MS3 scans and XlinkX 10-20-0.5 denotes MS1 and MS2 tolerances of 10/20 ppm and MS3 tolerance of 0.5 Da. MS Annika 2.0 outperforms all other tools at 1% and 5% experimentally validated FDR. At 1% experimentally validated FDR MS Annika 2.0 detects 7 times as many crosslinks as MaXLinker and 25% more than XlinkX. At 5% experimentally validated FDR MS Annika 2.0 provides 5 times as many crosslinks as MaXLinker and 16% more than XlinkX. MS Annika 2.0 also yields accurate FDR estimates, very close to the experimentally validated values for both 1% and 5% FDR results. FDR estimation of XlinkX is a lot better for this dataset, yielding experimentally validated FDRs up to 6.92% at 1% estimated FDR - still noticeably worse than MS Annika 2.0, however better than for the other datasets.

Supplementary Figure S8

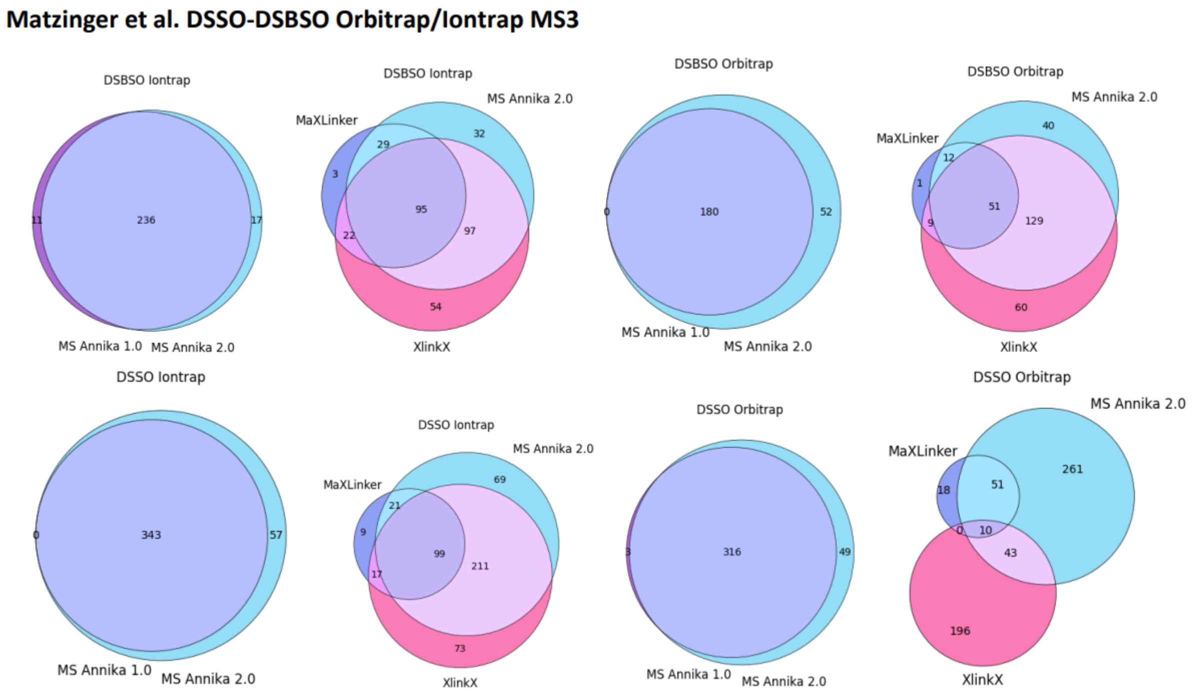

Fig. S8: *Dataset A* - Venn diagrams showing the overlaps of correctly identified crosslinks across the different search engines at 1% estimated FDR. Generally there is good agreement between the different tools, except for the DSSO Orbitrap dataset where sets of crosslinks identified by MS Annika 2.0/MaXLinker and XlinkX are quite disjoint. MS Annika 2.0 provides (almost) all crosslinks of MS Annika 1.0, except for the DSBSO Iontrap dataset where the difference is most likely due to different FDR cut-offs.

Supplementary Figure S9

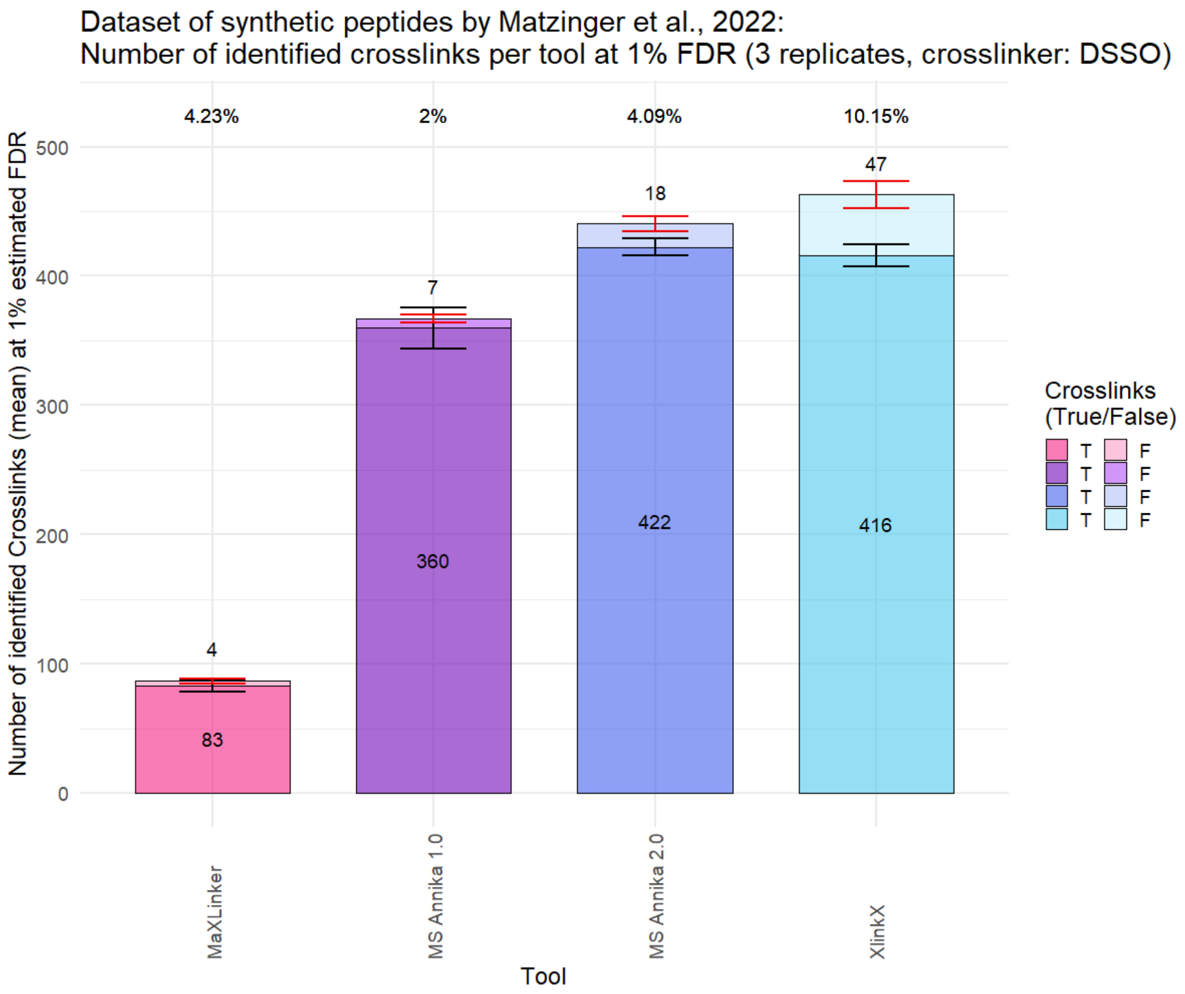

Fig. S9: Number of identified crosslinks and experimentally validated FDR of the different search engines at 1% estimated FDR for *dataset B*. True crosslinks are coloured in the darker shade and false crosslinks in the lighter shade. Numbers above the bar show the experimentally validated FDR. All numbers are averages from three technical replicates, the bars denote the standard deviation (black for true crosslinks, red for false crosslinks). MS Annika 2.0 outperforms MaXLinker and XlinkX in both the number of identified true crosslinks, reporting 5 times as many crosslinks as MaXLinker and slightly more than XlinkX, while also providing a more accurate FDR estimation of 4.09% experimentally validated FDR compared to 4.23% for MaXLinker and 10.15% for XlinkX.

Supplementary Figure S10

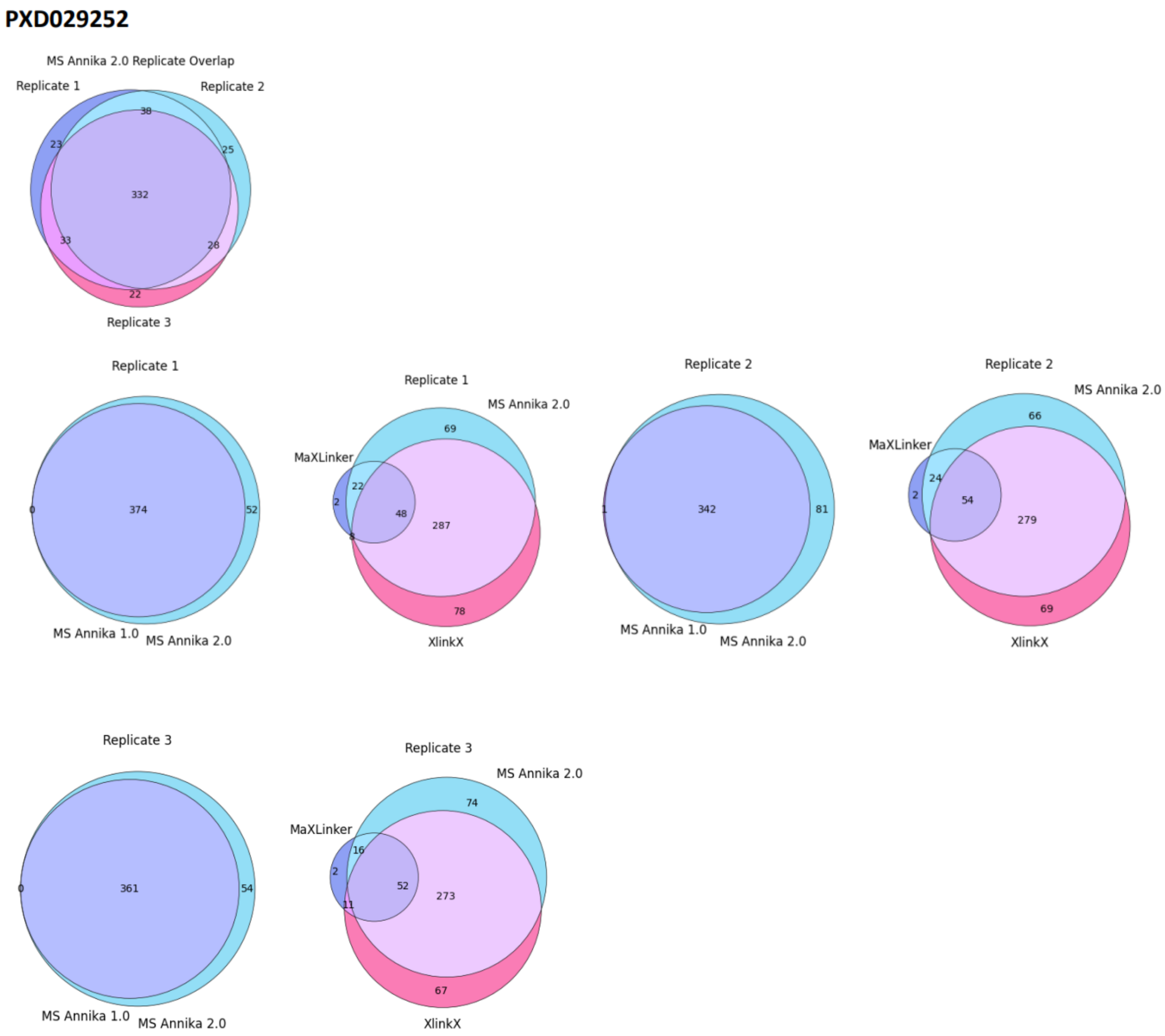

Fig. S10: *Dataset B* - Venn diagrams showing the overlaps of correctly identified crosslinks at 1% estimated FDR across the three replicates and the different search engines. There is good agreement between the replicates and the different tools. Again MS Annika 2.0 provides (almost) all crosslinks identified by MS Annika 1.0.

Supplementary Figure S11

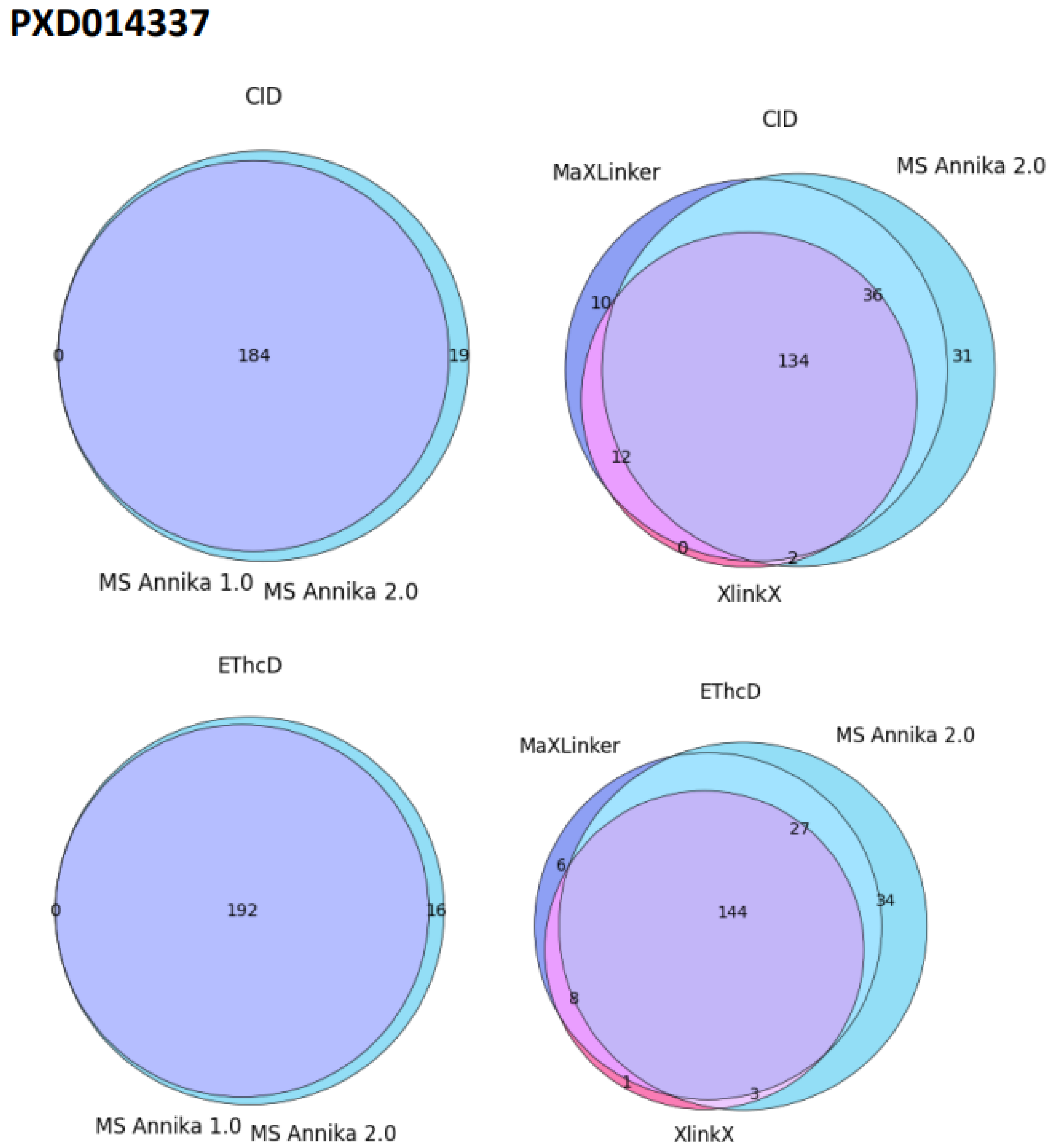

Fig. S11: *Dataset C* - Venn diagrams showing the overlaps of correctly identified crosslinks at 1% estimated FDR across the different search engines. The top two venn diagrams show overlaps for the CID-MS2-CID-MS3 experiment while the bottom two venn diagrams show overlaps for the CID-MS2-EThcD-MS2-CID-MS3 experiment. Generally there is good agreement between the different search tools and once again MS Annika 2.0 also provides all crosslinks identified by MS Annika 1.0.

Supplementary Figure S12

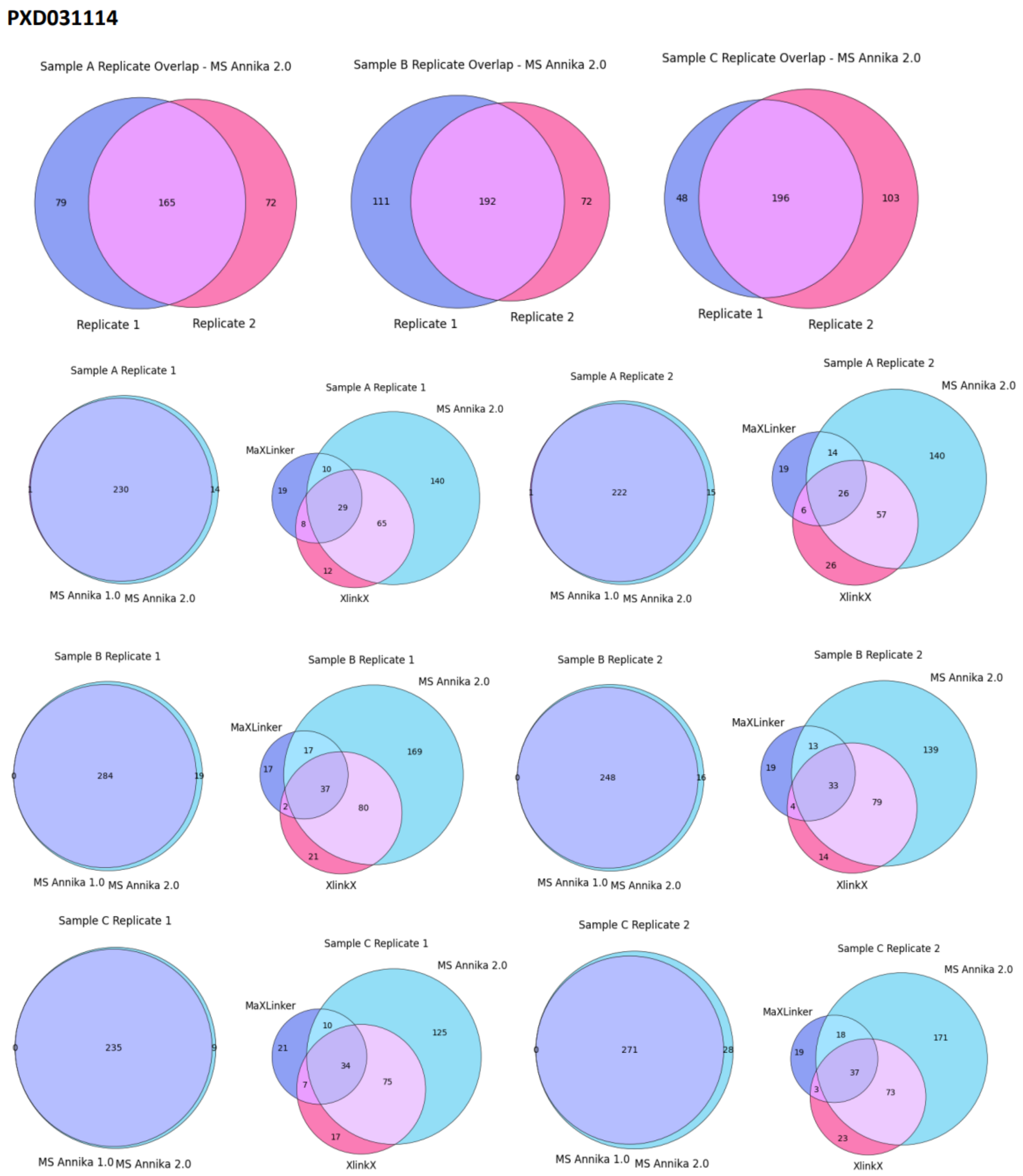

Fig. S12: *Dataset D* - Venn diagrams showing the overlaps of identified crosslinks at 1% entrapment FDR across the two replicates and the different search engines for all three SCX fractions. There is good agreement between the replicates and the different search tools. Again MS Annika 2.0 provides (almost) all crosslinks identified by MS Annika 1.0.

Supplementary Figure S13

MS Annika 2.0 crosslinks mapped to the NSP7-NSP8 complex

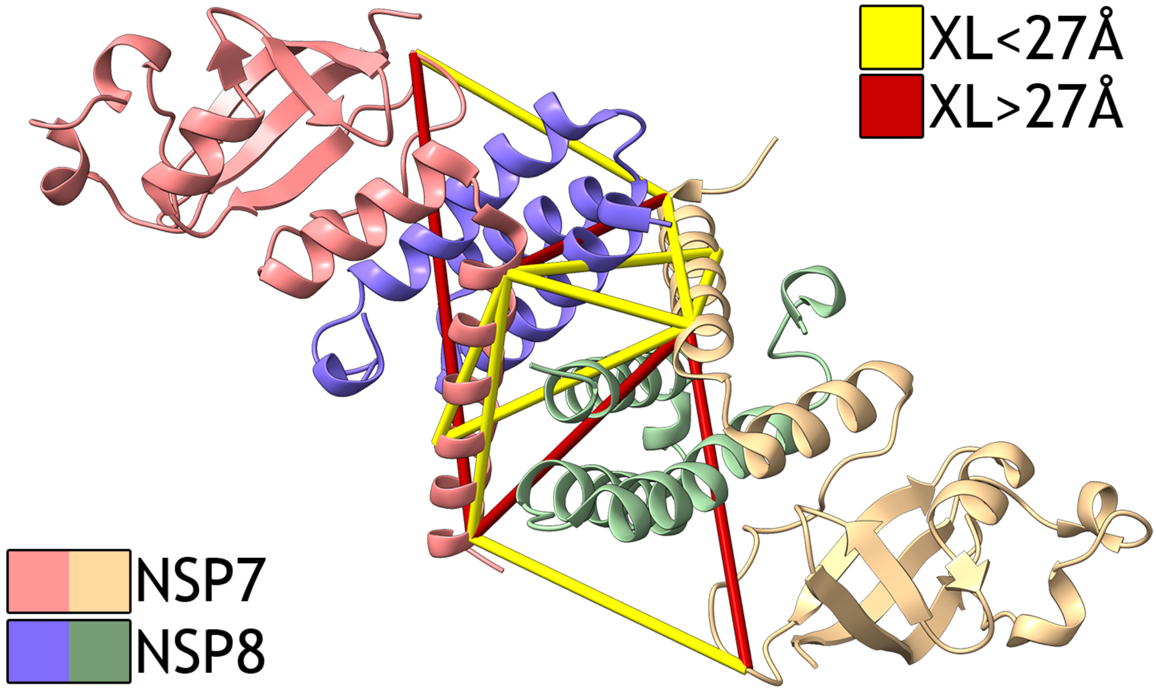

Fig. S13: *Dataset E* - Crosslinks uniquely identified by MS Annika 2.0 mapped to the 3D structure of the NSP7-NSP8 complex (PDB identifier 6YHU). Cross-linked residues within the 27 Å distance threshold of DSSO are coloured in yellow, cross-linked residues violating this threshold are coloured in red. Of the 17 additional crosslinks found by MS Annika 2.0, four could be mapped to the structure while the other 13 were corresponding to peptides that were not resolved in the complex. Due to the multimeric structure of the NSP7-NSP8 complex, three of the four crosslinks are ambiguous resulting in 13 cross-linked residues as seen in the figure in yellow and red. We could find cross-linked residues within the 27 Å distance constraint of DSSO for all four crosslinks reported by MS Annika 2.0, confirming their validity.

Supplementary Figure S14

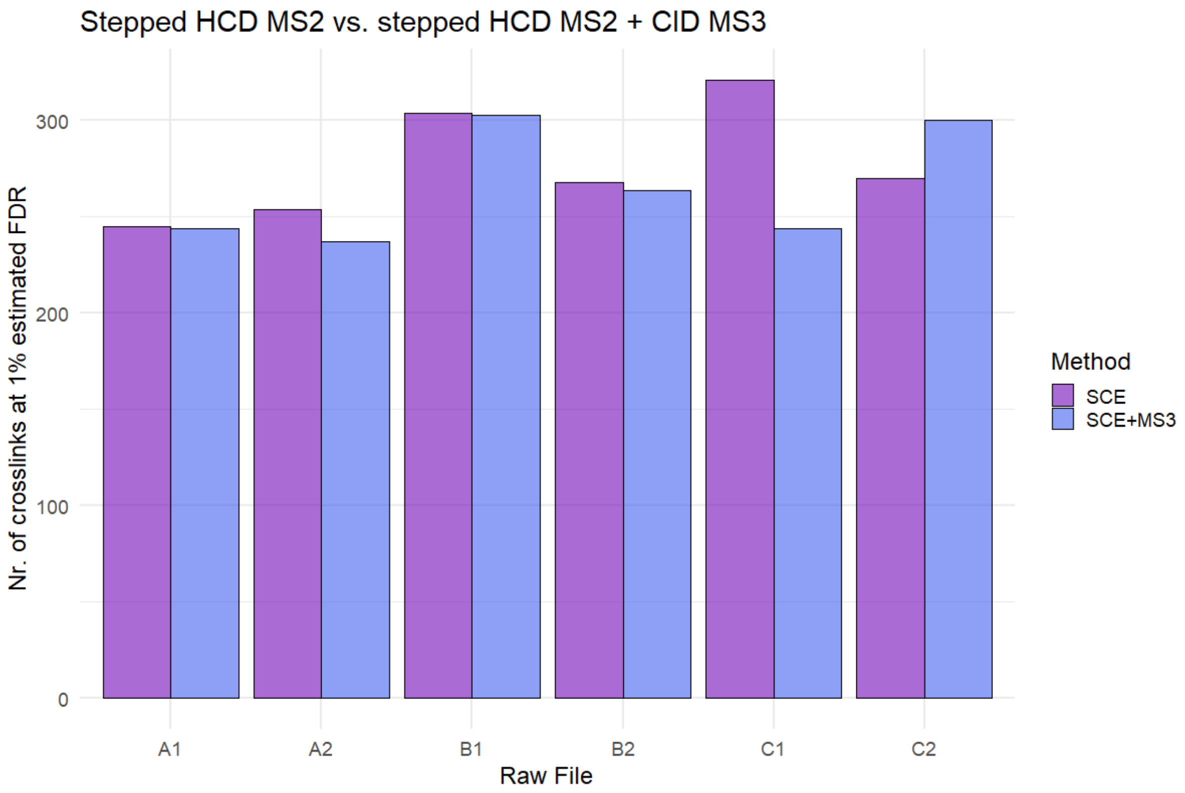

Fig. S14: *Dataset D (SCE: stepped HCD MS2 dataset, SCE+MS3: stepped HCD MS2 + CID MS3 dataset)* - Number of crosslinks identified by MS Annika 2.0 for the two different acquisition approaches across the three SCX fractions (A, B, C) and the two technical replicates (1, 2). The SCE dataset was analysed using the MS2 search of MS Annika 2.0 (which is identical to the MS2 search of the original MS Annika 1.0) and the SCE+MS3 dataset with the MS2-MS3 search approach. Overall more crosslinks can be identified when applying SCE-only acquisition which is likely due to shorter duty cycles, increasing scan rate and therefore sample coverage. However, SCE+MS3 is often only marginally behind or even in one case (C2) outperforming SCE. Considering the ongoing research in improving MS3 acquisition these results are promising that SCE+MS3 might potentially overtake SCE-only acquisition in the future.

References

[1] Athit Kao, Chi-li Chiu, Danielle Vellucci, Yingying Yang, Vishal R. Patel, Shenheng Guan, Arlo Randall, Pierre Baldi, Scott D. Rychnovsky, Lan Huang (2011) Development of a Novel Cross-linking Strategy for Fast and Accurate Identification of Cross-linked Peptides of Protein Complexes. Molecular & Cellular Proteomics, Volume 10, Issue 1. Doi: https://doi.org/10.1074/mcp.M110.002212
